# Estimating cumulative incidence from partially missing time series of respiratory viral infections

**DOI:** 10.1371/journal.pone.0353681

**Published:** 2026-09-15

**Authors:** Sudhir Venkatesan, Lisa White, Hyewon Nina Koo, John Dickerson, Weiming Hu, Jennifer Kuntz, Mark Schmidt, Sylvia Taylor, Carla Talarico

**Affiliations:** 1 BPM Evidence Statistics, BioPharmaceuticals Medical, AstraZeneca, Cambridge, United Kingdom; 2 Model Health Ltd, Brixham, United Kingdom; 3 Data Science and Artificial Intelligence, BioPharmaceuticals R&D, AstraZeneca, Mölndal, Sweden; 4 Kaiser Permanente Center for Health Research, Portland, Oregon, United States of America; 5 Medical Evidence, Vaccines and Immune Therapies, BioPharmaceuticals Medical, AstraZeneca, Cambridge, United Kingdom; 6 Medical Evidence, Vaccines and Immune Therapies, BioPharmaceuticals Medical, AstraZeneca, Gaithersburg, Maryland, United States of America; Università degli Studi di Parma: Universita degli Studi di Parma, ITALY

## Abstract

**Background:**

Seasonal respiratory viruses, including respiratory syncytial virus (RSV) and human metapneumovirus (hMPV), are major contributors to global respiratory infection burden. Estimating viral incidence using real-world data (RWD) is challenging as misalignment of data collection with seasonal circulation can lead to underestimated incidence and hinder inter-study comparisons.

**Methods:**

We developed a regression-based approach to retrospectively correct partial-season time series with contiguous missing weeks at the start or end of a season. Weekly RSV clinical incidence (per 100,000) was extracted from U.S. RWD sources (Optum^®^ CDM and PharMetrics Plus, adults ≥65 years; Kaiser Permanente Northwest [KPNW], all ages) for seasons 2018–19 to 2023–24 and aligned to RSV‑Net hospitalization surveillance. Weekly early- and late-season missingness scenarios were created (approximately 10%, 15%, 20%, and 25% of weeks removed). Models were fit within each season using harmonic terms and the surveillance-aligned covariate. Uncertainty was quantified using moving block bootstrap 95% prediction intervals. Weekly accuracy was evaluated using RMSE and normalized RMSE, and seasonal burden reconstruction accuracy using cumulative percent error and signed difference. For hMPV, we evaluated an illustrative extension using Optum^®^ CDM (adults ≥65 years; 2022–23 and 2023–24) and NREVSS test positivity; because positivity is bounded and depends on testing volume, we used a denominator-aware binomial/logit model (positives out of tests).

**Results:**

For RSV, weekly prediction error increased with missingness level, while reconstructed seasonal burden generally remained close to observed totals in applicable scenarios. Early-season gaps were associated with larger cumulative errors than late-season gaps in Optum^®^ CDM and PharMetrics Plus, whereas late-season median percent errors were closer to zero across missingness levels. Applicability was reduced primarily for higher early-season missingness in KPNW due to peak overlap (e.g., early 25% scenarios not applicable). For hMPV test positivity (illustrative; two seasons), weekly prediction RMSE was on the order of ~1–2 percentage points across missingness scenarios.

**Conclusion:**

Across multiple RSV seasons and data sources, reconstructed seasonal burden generally agreed with observed totals when missingness was confined to contiguous early- or late-season weeks and did not overlap peak activity. This approach provides a transparent method to improve the utility and comparability of partial-season respiratory virus time series. However, applicability is reduced when peak weeks are missing, and hMPV findings are illustrative given limited seasons and the use of test positivity.

## Introduction

Respiratory viral infections pose a significant global health challenge, contributing substantially to worldwide morbidity, mortality and economic disruption. In 2019, lower respiratory tract infections affected approximately 490 million individuals globally, resulting in 2.4 million deaths [1]. These infections disproportionately impact vulnerable populations, including immunocompromised individuals, children and the elderly, thereby presenting a critical public health concern. Viruses exhibiting seasonal patterns, such as influenza, respiratory syncytial virus (RSV) and human metapneumovirus (hMPV), are major contributors to this burden [2,3]. To monitor these viruses, several countries have implemented national, population-based and laboratory-based surveillance systems. While these systems provide valuable insights into national trends, assessing incidence within specific populations, healthcare settings or local regions typically necessitates prospective, multi-seasonal studies.

A primary challenge in designing such studies lies in accurately aligning the study start date with the onset of the respiratory virus season. This challenge is amplified when assessing multiple viruses simultaneously, as their seasonal patterns and peaks can vary by up to 3 months [4]. The COVID-19 pandemic further complicated this landscape by altering the seasonal patterns of circulating respiratory viruses in post-pandemic years [5]. Misalignment of study start dates with viral seasons can have important consequences. A delayed start could result in underestimated incidence due to missing early season data. Conversely, inconsistencies in defining a “season” can make it difficult to compare incidence estimates across studies [6]. This variability poses challenges for systematic reviews aiming to synthesize incidence estimates, as some studies report over truncated seasonal periods, missing late-season data, while others provide annual estimates over a longer timeframe. As a result, systematic reviews on respiratory virus burden often exclude studies with seasonal or partial-season data, potentially omitting valuable information [7,8]. This exclusion is particularly problematic for underreported viruses such as hMPV, where it further depletes an already sparse evidence base. Despite the frequency of partial-season reporting, there is limited guidance on how to standardize cumulative seasonal burden estimates across studies with asynchronous observation windows, especially using real-world data sources whose observation periods may not align with seasonal circulation.

To address these challenges, we propose a transparent, reproducible method that uses known seasonality and surveillance-aligned dynamics to correct contiguous early- or late-season gaps and estimate cumulative RSV and hMPV incidence. In this work, we demonstrate the application of this method and retrospectively assess its accuracy across several seasons using three distinct data sources from the United States (US). Our approach aims to enhance the utility of partial-season data to further our understanding of seasonal respiratory viruses.

## Methods

### Data sources

Two types of data were required for this analysis: surveillance data to represent national/regional disease dynamics, and study-specific data that are typically obtained either from a study site or from a large real-world data (RWD) network. For surveillance network data, the following sources were used:

- **RSV-Net**: a population-based surveillance system established by the US Centers of Disease Control and Prevention (CDC), within the Respiratory Virus Hospitalization Surveillance Network (RESP-NET), to monitor RSV-associated hospitalizations [9]. RSV-Net provides weekly incidence data (per 100,000 populations) from the 2018–19 season onwards, stratified by age (at the national level only), sex, race/ethnicity and state.
- **The National Respiratory and Enteric Virus Surveillance System (NREVSS)**: a laboratory surveillance system also established by the US CDC [10]. NREVSS reports weekly test-positivity proportions, both nationally and by US region (as defined by the US Department of Health and Human Services), for a range of respiratory and enteric viruses from participating laboratories. For the work described in this manuscript, we used the hMPV test-positivity data which have been available since the 2019–20 season.

RSV-Net reports hospitalization incidence, whereas the study outcomes derived from claims/EHR reflect diagnosis, and/or lab-confirmed clinical events, and therefore differ in clinical severity and ascertainment processes. In this work, surveillance is used as a temporal anchor for epidemic dynamics (timing and seasonal intensity) rather than as a direct measurement of the same outcome. We fit models within each season so that the surveillance-to-study mapping is estimated from the season’s observed overlap rather than assumed constant across years.

For the RWD component, three data sources were included:

- **Optum’s de-identified Clinformatics**^**®**^ **Data Mart Database (accessed 24 December 2024, and referred to as Optum**^®^ **CDM henceforth)**: a large, de-identified US administrative claims database that captures patient-level medical and pharmacy claims, laboratory testing and results, and enrolment data. It is derived from a database of administrative health claims for members of large commercial and Medicare Advantage health plans, covering over 60 million individuals [11,12].
- **IQVIA PharMetrics**^**®**^ **Plus (accessed 24 December 2024, and referred to as PharMetrics Plus henceforth)**: a large, longitudinal US administrative claims database that provides integrated medical and pharmacy claims data for over 190 million unique patients. It captures information from commercial health plans and self-insured employer groups across the USA. The database includes detailed patient demographics, diagnoses, procedures, prescription drug information and healthcare utilization data.
- **Kaiser Permanente Northwest (accessed 22 January 2025, and referred to as KPNW henceforth)**: a comprehensive electronic health record (EHR) system database that captures detailed clinical and administrative data for approximately 600,000 members in the states of Oregon and Washington. It includes longitudinal patient records with rich clinical information such as diagnoses, procedures, laboratory testing and results, prescription medication dispensing and healthcare utilization.

We extracted aggregated weekly RSV incidence (per 100,000 population) from each of the three RWD sources, and hMPV test-positivity proportions from Optum^®^ CDM. RSV and hMPV infection status was ascertained based on the presence of a relevant diagnostic code or positive laboratory test for Optum^®^ CDM and KPNW, and using diagnostic codes only for PharMetrics Plus. A listing of the diagnostic codes utilized in this analysis is provided in S1 Table. Due to the nature of the data extraction, no information that could be used to identify individuals was obtained for these analyses. It is important to note that the intention behind including three sources for RWD was not to perform a direct comparison between them or to assess their suitability for studies of RSV or hMPV incidence. Rather, our aim was to evaluate the performance of our linear regression-based cumulative incidence estimation method on three data sources with different properties, patient profiles and levels of diagnostic certainty.

### Study populations and seasons

The study populations comprised adults ≥65 years of age for Optum^®^ CDM and for PharMetrics Plus, given the disproportionate impact of RSV infections in this age group. Correspondingly, we used weekly national RSV incidence from RSV-Net for the same age group within these two data sources. Since RSV-Net does not report age-stratified incidence for Oregon, where KPNW is based, the study population for KPNW did not have an age restriction, and all age groups were included. Therefore, the weekly RSV incidence for Oregon across all age groups was used for this analysis. RSV seasons, as defined by the US CDC, from 2018–19 to 2023–24 were included for Optum^®^ CDM and PharMetrics Plus; because of sparse data in previous seasons, only seasons from 2021–22 to 2023–24 were included for KPNW.

The hMPV analyses were based on testing data obtained from adults ≥65 years of age in Optum^®^ CDM. This was the only data source that was included in the hMPV analysis, as PharMetrics Plus does not report laboratory tests and the data from KPNW were too sparse. Correspondingly, we used hMPV test-positivity data from NREVSS for this analysis. We only included hMPV seasons 2022–23 and 2023–24 in our analysis.

### Statistical analyses

Our objective is retrospective correction of partial seasonal series, not real‑time nowcasting or forecasting. We consider two settings: (i) early‑season gaps (e.g., delayed start), where the remainder of the season is observed, and (ii) late‑season gaps (e.g., early termination), where the beginning of the season is observed. Accordingly, statistical analyses were conducted using the three RWD data sources as follows:

1. For corresponding time periods, we extracted weekly time series from the surveillance and RWD sources on incidence (for the RSV analysis) and test-positivity proportions (for the hMPV analysis).
2. Data were then split by season (as defined by the US CDC). The 2018–19 and 2019–20 seasons are reported as 31-week seasons by RSV-Net, extending from MMWR (Morbidity and Mortality Weekly Report) Week 40 to Week 18 in the subsequent year. All remaining seasons are reported in RSV-Net as 52-week seasons extending from Week 40 to Week 39 in the subsequent year. For Optum^®^ CDM and PharMetrics Plus, the 2023–24 season only had data through Weeks 37 and 24, respectively, based on the latest data extract available to us at the time of conducting these analyses.
3. Scenarios with early- and late-season missingness were created by removing portions of incidence/test-positivity data from the start and end of each season, respectively. Four levels of missingness were modelled: approximately 10%, 15%, 20% and 25% of total available weeks per season. This corresponded to:
  - For 31-week seasons: 3, 5, 6 and 8 weeks
  - For 52-week seasons: 5, 8, 10 and 13 weeks
  - For the 2023–24 season:
    - Optum^®^ CDM: 4, 6, 7 and 9 weeks
    - PharMetrics Plus: 2, 4, 5 and 6 weeks

For instance, in a 52-week season, the first 8 weeks were removed for 15% early-season missingness, and separately, the last 13 weeks for 25% late-season missingness. If the omitted block overlapped the seasonal peak week (defined as the week with maximum observed incidence within that season), the scenario was classified as not applicable and no prediction was attempted. We report the frequency of such peak-overlap scenarios (applicability rate) by source, season, and missingness level. Reported performance metrics are out-of-sample within season: for each season and scenario, models were fit only on the observed weeks (training segment) and evaluated on the withheld contiguous block (holdout segment). Metrics are not in-sample goodness-of-fit.

4. To each of the above incomplete seasons, we fit a linear regression model as follows:

$$\begin{array}{c} {Weekly\;estimate}_{RWD}=\beta_{\text{0}}+\beta_{\text{1}}{Weekly\;estimate}_{surveillance}+\beta_{\text{2}}\text{sin}\left(\frac{\text{2}\pi t}{\text{5}\text{2}.\text{1}\text{8}}\right)+\beta_{\text{3}}\text{cos}\left(\frac{\text{2}\pi t}{\text{5}\text{2}.\text{1}\text{8}}\right)+\varepsilon \end{array}$$

Where β_0_ to β_3_ represent coefficients associated with the intercept and each of the independent variables in the model; t indexes week number; ε is the error term; and the sine and cosine terms represent seasonal components with weekly periodicity. For each missingness scenario, Weekly estimate_surveillance_ was standardized using the training segment only (mean 0, standard deviation 1) before model fitting.

5. Using the above linear regression model fit, weekly incidence of RSV/test-positivity proportions for hMPV for the missing weeks, with 95% prediction intervals (95% PI), were predicted. This generated a reconstructed full season, combining predicted values for missing weeks with observed values for the remaining weeks. For the RSV analyses, the weekly incidence data were used to estimate the cumulative reconstructed incidence across an entire season.

Our regression model for RSV was specified as follows:

**Time index and seasons**: Let t denote MMWR week within a CDC‑defined respiratory season (detailed earlier in this section). Analyses are performed within season, with t reset to 1 at each season’s start.

**Period choice**: Seasonality was represented using a single harmonic function (sine and cosine terms) with period P = 52.18 weeks to reflect 365.25 days/year. This choice was made a priori based on domain knowledge and prior epidemiologic applications of harmonic regression for seasonal infectious diseases, and to maintain a parsimonious model given the limited number of observations per season. A *post hoc* sensitivity analysis using P = 52 was performed to assess the robustness of our period choice.

**Outcomes and units**: For RSV, the outcome y_t is weekly incidence per 100,000 population derived from each RWD source. No variance‑stabilizing transforms were applied. To respect natural bounds, RSV weekly predictions are truncated at 0 before aggregation (non‑negative incidence). We report the frequency of negative weekly predictions as a diagnostic.

**Error structure and prediction intervals**: As weekly residuals were likely to be autocorrelated, 95% PI for held-out weeks were constructed using a moving block bootstrap (MBB) of model residuals. Specifically, we resampled contiguous residual blocks from the training segment and added these to the mean predictions to obtain an empirical predictive distribution for each omitted week, from which 2.5th and 97.5th percentiles were taken as the 95% PI. We additionally report Ljung–Box tests of residual autocorrelation and empirical 95% PI coverage on the held-out weeks. The MBB 95% PI were implemented as follows:

- We constructed 95% weekly and cumulative PI using a MBB on the per‑season Ordinary Least Squares (OLS) residuals from the observed segment (training weeks only) using 2000 bootstrap replicates.
- Block length: l = max(2, round(n_train^(1/3))) with an upper cap of floor(0.5 × n_train); this adapts to the available training length while preserving short‑lag dependence.
- Resampling scheme: From the residual vector (length n_train), we formed all contiguous blocks of length l and sampled k = ceiling(n_train/l) blocks with replacement; the concatenated series was truncated to length n_train (no circular wrapping). Residuals were not re‑centered (the fitted model includes an intercept; mean residual ≈ 0).
- Refitting and coefficient uncertainty: For each replicate, we added the resampled residual series to the OLS fitted values on the training weeks to create y*, re‑fit the same per‑season regression, and generated predictions for the omitted block. This procedure propagates parameter uncertainty through re‑estimation each bootstrap.
- Non‑negativity: Weekly bootstrap predictions were truncated at zero before summarization.
- Weekly bands: The 2.5^th^ and 97.5^th^ percentiles across B predictions at each omitted week defined the weekly 95% PI, preserving week‑to‑week correlation via block resampling.
- Cumulative bands: For each bootstrap path, we formed the cumulative sum of the predicted omitted block and added the observed part of the season (head for late gaps; tail for early gaps). Pointwise 2.5^th^/97.5^th^ percentiles across B provided cumulative ribbons; the same bootstrap totals yielded 95% intervals for the reconstructed seasonal total.
- Assumptions and limitations: The MBB assumes weak stationarity of residuals over the training segment. Near steep early ramps/peaks local variance can be higher and training segments shorter; consistent with this, empirical weekly coverage declines at higher early‑season missingness, whereas late‑season coverage is high.

**Fitting protocol and validation splits:** Models were fit separately by season (no pooling across seasons). Validation used a within-season block holdout design: contiguous early-season or late-season weeks were removed (10%, 15%, 20%, 25% of weeks, per season length), predicted using the fitted model, and compared against observed values. If the withheld block overlapped with the season’s peak week, the scenario was classified as not applicable and no prediction was attempted. To assess whether the surveillance-to-study relationship was stable across seasons, we summarized season-by-season fitted coefficients and overlap correlations between the RWD data and the surveillance report on the observed segment.

Our primary outcome was the season‑long cumulative incidence for RSV, obtained by summing weekly incidence over the reconstructed series (observed weeks plus model‑predicted values for the omitted block).

Weekly predictive accuracy was assessed using root mean squared error (RMSE) on held-out weeks and normalized RMSE (nRMSE), defined as RMSE divided by the season’s mean weekly incidence to facilitate comparison across seasons with different magnitudes. For seasonal burden, we assessed reconstruction accuracy using cumulative percent error, signed difference (per 100,000), and log-ratio error (using a small constant to avoid division by zero when totals were very small). We then summarized results as median (IQR) across seasons and scenarios, with bootstrap 95% CI for medians.

All model development and diagnostics (weekly RMSE, weekly 95% PI with moving‑block bootstrap, and empirical coverage) are reported to contextualize uncertainty at the week level, but decision‑relevant accuracy is evaluated at the cumulative, seasonal level. We quantify seasonal accuracy using percent relative error, defined as [(reconstructed cumulative incidence ÷ observed complete‑season cumulative incidence) − 1] × 100, and also report the signed difference per 100,000 and the log‑ratio error to provide scale‑dependent and scale‑free perspectives. Because aggregation attenuates week‑to‑week noise and we truncate any negative weekly incidence predictions at 0 before summation. For transparency, each scenario also reports the proportion of cases missing (observed cases in the omitted block divided by the observed complete‑season total), which better reflects reconstruction difficulty than the proportion of weeks missing alone and explains asymmetry in errors across seasons with the same share of weeks removed. As a sensitivity analysis, we repeated all across‑season summaries after excluding the truncated 2023–24 season (Optum^®^ CDM through Week 37; PharMetrics Plus through Week 24) to ensure pooled summaries were not driven by an incomplete reference year. As a robustness check, we re‑fit the per‑season model with a non‑negative link (quasi‑Poisson with log link and log‑population offset where counts were available; otherwise, gamma Generalized Linear Model [GLM] with log link on rates) using the same covariates, and compared reconstructed cumulative totals with OLS for ≈20% early‑season gaps across all sources and seasons. Importantly, we model the RWD outcome (diagnosis and/or lab‑confirmed clinical events per 100,000) and use RSV‑Net hospitalization rates as a temporal covariate. We assume temporal alignment, not equality of levels. The estimand is the season‑long cumulative total of the RWD outcome.

For hMPV, nationally available incidence surveillance analogous to RSV-Net was not available. Therefore, we evaluated the framework using weekly hMPV test positivity from CDC NREVSS as the surveillance-aligned signal and weekly hMPV test positivity in Optum^®^ CDM (adults ≥65 years) as the study outcome. We modified the outcome model to respect the bounded nature of positivity and to account for varying precision across weeks. Specifically, we modelled weekly counts of positive tests out of total tests using a binomial/logit specification, with the surveillance positivity entered as a standardized covariate computed on the training segment as follows:

For week *t* in season *s*, let *n_test*,*s*,*t* be the number of hMPV tests performed and *n_pos,s,t* the number positive. We model *n_pos,s,t | n_test,s,t ~ Binomial(n_test,s,t, p_s,t) with logit(p_s,t) =* β*0,s +* β*1,s x_std,s,t +* β*2,s sin(2*π *t/P) +* β*3,s cos(2*π *t/P),* where P = 52.18 (weeks)

Surveillance covariate: *x_s,t* is the contemporaneous NREVSS hMPV positivity (proportion) aligned to the same calendar week; *x_std,s,t = (x_s,t − mean_train(x_s,·))/sd_train(x_s,·)* is standardized using the training segment for that scenario.

We fit a quasi‑binomial GLM with logit link to allow overdispersion. Weekly 95% PIs were generated using an MBB to account for serial correlation, and performance was assessed on held-out weeks under early- and late-season missingness scenarios.

All analyses were conducted in R (version 4.5.1). Annotated R code demonstrating the RSV and hMPV imputation workflows on simulated data is provided in the Supporting Information.

### Ethics

KPNW received approval from their interregional institutional review board, KPiLRB, to carry out this work. Optum^®^ CDM and PharMetrics Plus use did not qualify for institutional review board/ethics committee approval as all data access was granted under existing data use agreements, was conducted in line with the terms of those agreements, and did not contain any patient identifiable information that would result in a classification as human subjects research. All work used only retrospective, de-identified data in compliance with the Health Insurance Portability and Accountability Act standards. No direct patient contact occurred, and no identifiable private information was used. All analyses were conducted in accordance with the ethical principles originating from the Declaration of Helsinki and were consistent with the International Council for Harmonisation/Good Clinical Practice, applicable regulatory requirements, and applicable legislation on non-interventional studies/observational studies.

## Results

### RSV incidence

We evaluated a regression-based approach to retrospectively correct partially observed RSV seasons by reconstructing contiguous early-season or late-season gaps and recovering seasonal cumulative clinical incidence. Analyses included six U.S. RSV seasons (2018–19 to 2023–24) in Optum^®^ CDM and PharMetrics Plus (adults ≥65 years) and three seasons (2021–22 to 2023–24) in KPNW (all ages). Across sources, season timing and magnitude varied substantially across years, including atypical dynamics in pandemic-disrupted seasons.

### Weekly reconstruction accuracy and applicability

Weekly prediction performance is summarized in Table 1 as median (IQR) RMSE and normalized RMSE (nRMSE) across seasons, together with empirical 95% PI coverage and an applicability rate. As intended, prediction was not attempted for scenarios in which the omitted block overlapped the seasonal peak (“peak reached”); this was most frequent for higher early-season missingness in KPNW (e.g., early 25% scenarios were not applicable for all KPNW seasons; Table 1). Across applicable scenarios, weekly error generally increased with missingness level. Empirical 95% PI coverage was high for most 10–20% missingness scenarios but declined in the most challenging settings (notably 25% missingness in Optum^®^ CDM and PharMetrics Plus; Table 1).

**Table 1:** Weekly reconstruction performance and applicability for RSV clinical incidence (P = 52.18).

Data source	Missingness location (Early-season / Late-season)	Missingness level (10%, 15%, 20%, 25% of weeks)	Seasons evaluated (n)	Applicable scenarios (n)	Applicability rate	Weekly RMSE, median (IQR)	Weekly nRMSE, median (IQR)	Empirical 95% PI coverage, median (IQR)
Optum	early	10%	6	6	1	0.506 (0.305)	0.199 (0.065)	1.000 (0.000)
Optum	early	15%	6	6	1	0.735 (0.477)	0.250 (0.098)	0.938 (0.181)
Optum	early	20%	6	6	1	0.781 (0.980)	0.355 (0.272)	0.917 (0.451)
Optum	early	25%	6	6	1	1.316 (2.796)	0.746 (0.298)	0.519 (0.356)
Optum	late	10%	6	6	1	0.499 (0.337)	0.217 (0.158)	1.000 (0.150)
Optum	late	15%	6	6	1	0.455 (0.372)	0.199 (0.169)	0.900 (0.238)
Optum	late	20%	6	5	0.833	0.506 (0.434)	0.148 (0.123)	1.000 (0.100)
Optum	late	25%	6	5	0.833	0.695 (0.606)	0.257 (0.309)	0.923 (0.500)
Pharmetrics	early	10%	6	6	1	0.592 (0.130)	0.278 (0.110)	1.000 (0.150)
Pharmetrics	early	15%	6	6	1	0.868 (0.297)	0.437 (0.139)	0.838 (0.206)
Pharmetrics	early	20%	6	6	1	0.958 (0.471)	0.522 (0.197)	0.683 (0.258)
Pharmetrics	early	25%	6	6	1	1.020 (2.141)	0.698 (0.635)	0.442 (0.248)
Pharmetrics	late	10%	6	5	0.833	0.853 (0.347)	0.203 (0.311)	1.000 (0.400)
Pharmetrics	late	15%	6	5	0.833	0.883 (0.640)	0.255 (0.420)	1.000 (0.500)
Pharmetrics	late	20%	6	5	0.833	0.977 (0.393)	0.471 (0.261)	0.833 (0.457)
Pharmetrics	late	25%	6	5	0.833	1.247 (0.763)	0.649 (0.147)	0.375 (0.359)
KPNW	early	10%	3	3	1	1.248 (1.235)	0.430 (0.124)	1.000 (0.000)
KPNW	early	15%	3	2	0.667	1.119 (0.178)	0.328 (0.119)	0.938 (0.062)
KPNW	early	20%	3	2	0.667	2.364 (0.122)	0.662 (0.111)	0.800 (0.100)
KPNW	early	25%	3	0	0	NA	NA	NA
KPNW	late	10%	3	3	1	1.338 (1.052)	0.373 (0.063)	1.000 (0.000)
KPNW	late	15%	3	3	1	1.425 (1.024)	0.451 (0.095)	1.000 (0.062)
KPNW	late	20%	3	3	1	1.447 (1.110)	0.485 (0.089)	1.000 (0.050)
KPNW	late	25%	3	3	1	1.817 (1.126)	0.514 (0.133)	1.000 (0.077)

95% PI: 95% Prediction intervals; IQR: Interquartile range; NA: Not applicable; nRMSE: Normalized root mean squared error; RMSE: Root mean squared error; RSV: Respiratory syncytial virus.

### Cumulative seasonal burden accuracy (early-season missingness)

For early-season gaps, reconstructed cumulative incidence error increased with missingness level in Optum^®^ CDM and PharMetrics Plus (Table 2). In Optum^®^ CDM, median cumulative percent error increased from 1.36% (IQR 2.21) at 10% missingness to 13.02% (IQR 5.83) at 25%, with corresponding median signed differences increasing from 1.28 to 11.64 per 100,000. PharMetrics Plus showed a similar pattern (median −0.82% at 10% to 10.76% at 25%; Table 2). In KPNW, early-season performance was generally accurate at 10–15% missingness, while higher early-season missingness more frequently intersected peak activity and was therefore not applicable (Table 2; S7 Table).

**Table 2:** Seasonal cumulative reconstruction accuracy for RSV clinical incidence under early-season missingness (P = 52.18).

Data Source	Missingness level (10%, 15%, 20%, 25% of weeks)	Seasons evaluated (n)	Applicable scenarios (n)	Applicability rate	Cumulative percent error (%), median (IQR)	Cumulative signed difference (per 100,000), median (IQR)	Cumulative log-ratio error, median (IQR)
Optum	10%	6	6	1	1.36 (2.21)	1.28 (1.90)	0.013 (0.022)
Optum	15%	6	6	1	3.26 (3.59)	2.75 (2.58)	0.032 (0.035)
Optum	20%	6	6	1	5.13 (6.91)	5.96 (4.64)	0.050 (0.065)
Optum	25%	6	6	1	13.02 (5.83)	11.64 (22.82)	0.122 (0.051)
Pharmetrics	10%	6	6	1	−0.82 (4.50)	−0.89 (3.77)	−0.008 (0.045)
Pharmetrics	15%	6	6	1	0.24 (9.34)	−1.24 (9.74)	0.002 (0.092)
Pharmetrics	20%	6	6	1	3.89 (9.90)	2.62 (10.34)	0.038 (0.099)
Pharmetrics	25%	6	6	1	10.76 (9.46)	5.27 (3.97)	0.102 (0.087)
KPNW	10%	3	3	1	−0.07 (0.94)	−0.28 (1.52)	−0.001 (0.009)
KPNW	15%	3	2	0.667	0.17 (1.30)	−0.21 (2.43)	0.002 (0.013)
KPNW	20%	3	2	0.667	−7.52 (0.30)	−14.61 (3.73)	−0.078 (0.003)
KPNW	25%	3	0	0	NA	NA	NA

IQR: Interquartile range; NA: Not applicable; RSV: Respiratory syncytial virus.

### Cumulative seasonal burden accuracy (late-season missingness)

For late-season gaps, Optum^®^ CDM and PharMetrics Plus showed smaller median errors than early-season gaps at comparable missingness levels (Table 3, S7 Table, S2 File), consistent with late-missingness scenarios retaining the rise and peak in the observed segment. In Optum^®^ CDM, median cumulative percent error remained close to zero at 10–15% missingness (0.44% and −0.27%, respectively) and was modestly negative at higher missingness among applicable scenarios (Table 3). PharMetrics Plus late-season median errors were also small on average, with increasing variability at higher missingness (Table 3). In contrast, KPNW late-season reconstructions showed increasing overestimation with missingness level (median 2.97% at 10% to 12.18% at 25%; Table 3).

**Table 3:** Seasonal cumulative reconstruction accuracy for RSV clinical incidence under late-season missingness (P = 52.18).

Data Source	Missingness level (10%, 15%, 20%, 25% of weeks)	Seasons evaluated (n)	Applicable scenarios (n)	Applicability rate	Cumulative percent error (%), median (IQR)	Cumulative signed difference (per 100,000), median (IQR)	Cumulative log-ratio error, median (IQR)
Optum	10%	6	6	1	0.44 (2.84)	0.33 (2.88)	0.004 (0.028)
Optum	15%	6	6	1	−0.27 (2.49)	−0.52 (3.72)	−0.003 (0.025)
Optum	20%	6	5	0.833	−1.10 (1.30)	−2.06 (4.06)	−0.011 (0.013)
Optum	25%	6	5	0.833	−2.37 (6.86)	−5.90 (2.90)	−0.024 (0.073)
Pharmetrics	10%	6	5	0.833	1.89 (2.74)	2.75 (4.62)	0.019 (0.027)
Pharmetrics	15%	6	5	0.833	2.91 (6.16)	4.22 (8.44)	0.029 (0.061)
Pharmetrics	20%	6	5	0.833	2.94 (14.05)	4.26 (13.08)	0.029 (0.140)
Pharmetrics	25%	6	5	0.833	3.49 (22.65)	5.06 (17.23)	0.034 (0.225)
KPNW	10%	3	3	1	2.97 (0.68)	6.24 (5.66)	0.029 (0.007)
KPNW	15%	3	3	1	6.75 (1.30)	10.52 (8.13)	0.065 (0.012)
KPNW	20%	3	3	1	8.72 (1.58)	13.75 (11.14)	0.084 (0.015)
KPNW	25%	3	3	1	12.18 (2.98)	21.89 (13.88)	0.115 (0.027)

IQR: Interquartile range; NA: Not applicable; RSV: Respiratory syncytial virus.

### Season-by-season patterns and uncertainty

**Fig 1:**
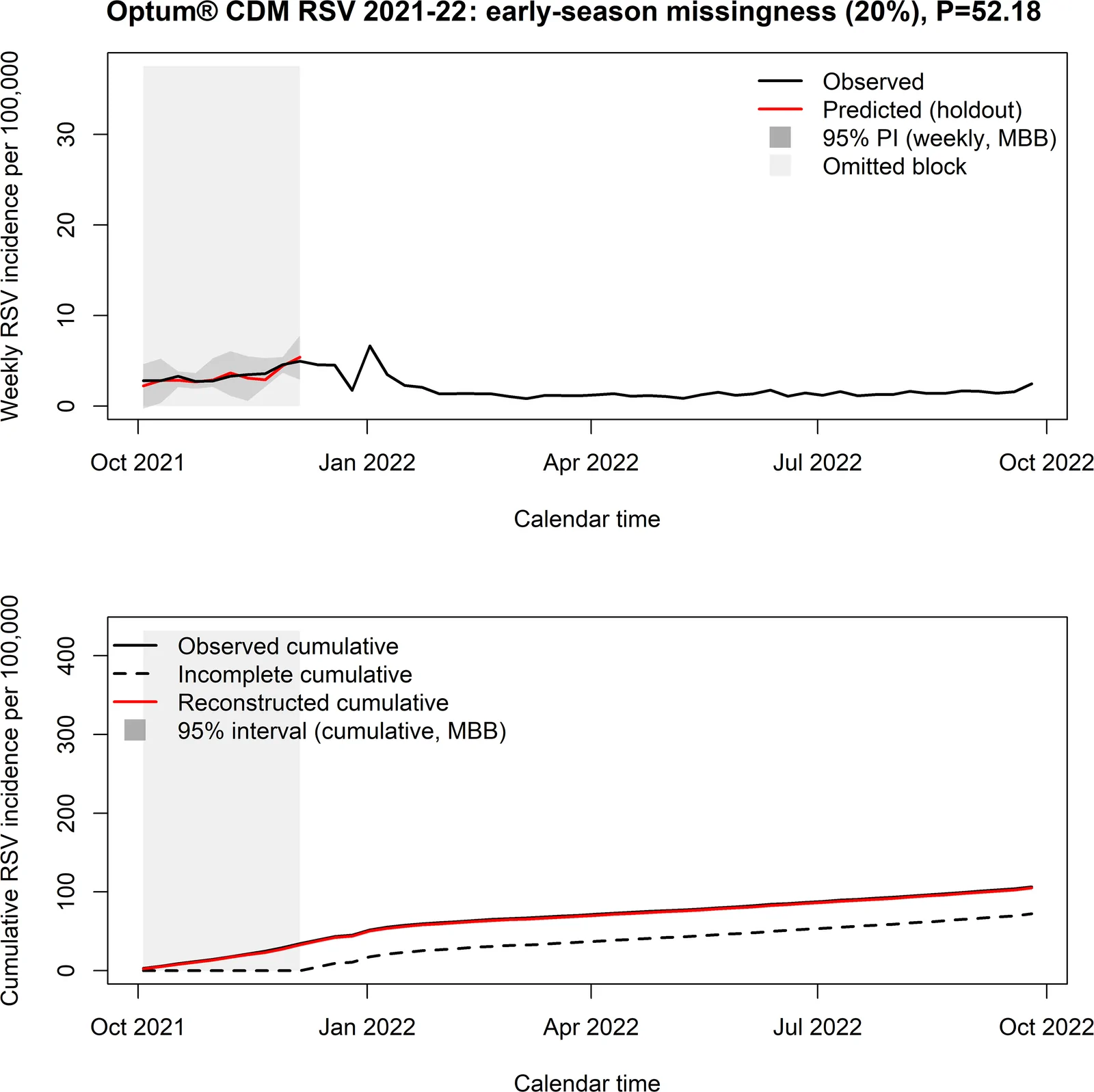
Model reconstruction of weekly (top) and cumulative (bottom) RSV clinical incidence for an early-season missingness scenario (Optum^®^ CDM, 2021–22 season). Weekly RSV clinical incidence (per 100,000) is shown as observed (black solid). The omitted block (early-season gap) is shaded. Red line denotes model-predicted weekly values for the omitted weeks; the grey band indicates 95% Prediction Intervals (PI) constructed using a moving block bootstrap (MBB) to account for serial correlation. In the cumulative panel, the black solid line is the observed cumulative incidence, the black dashed line shows the incomplete cumulative incidence that would be obtained by doing nothing (treating omitted weeks as missing/zero contribution), and the red line shows the reconstructed cumulative incidence after imputation; the grey band shows the corresponding MBB-based 95% interval for the cumulative reconstruction, displayed only from the start of the omitted block onward. RSV: Respiratory syncytial virus.

**Fig 2:**
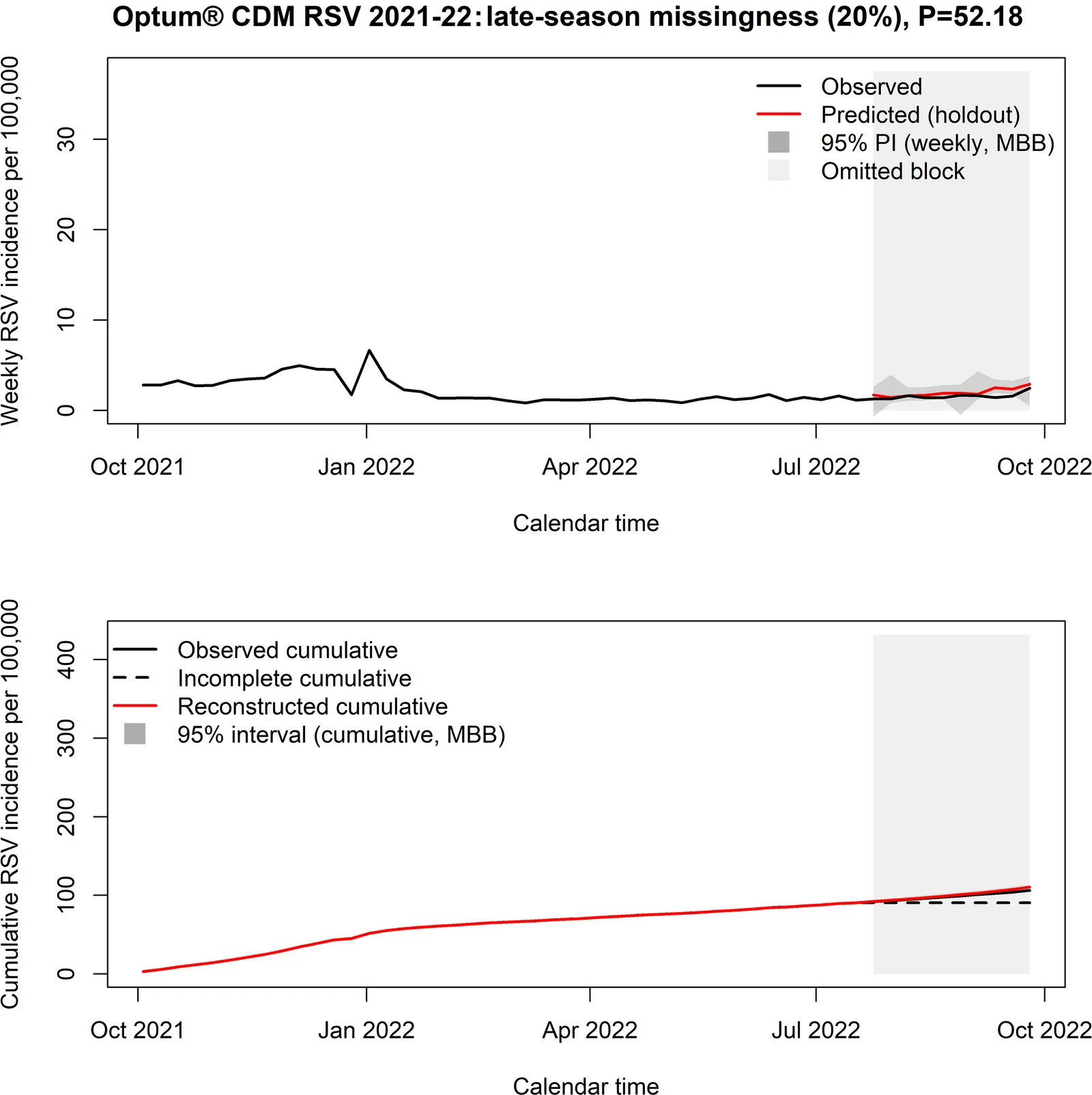
Model reconstruction of weekly (top) and cumulative (bottom) RSV clinical incidence for a late-season missingness scenario (Optum^®^ CDM, 2021–22 season). Weekly RSV clinical incidence (per 100,000) is shown as observed (black solid). The omitted block (late-season gap) is shaded. Red line denotes model-predicted weekly values for the omitted weeks; grey band indicates 95% MBB prediction intervals (PI). In the cumulative panel, the black solid line is the observed cumulative incidence, the black dashed line is the incomplete cumulative incidence (no imputation), and the red line is the reconstructed cumulative incidence after imputation; the grey band shows the MBB-based 95% interval for the cumulative reconstruction from the start of the omitted block onward. RSV: Respiratory syncytial virus.

**Fig 3:**
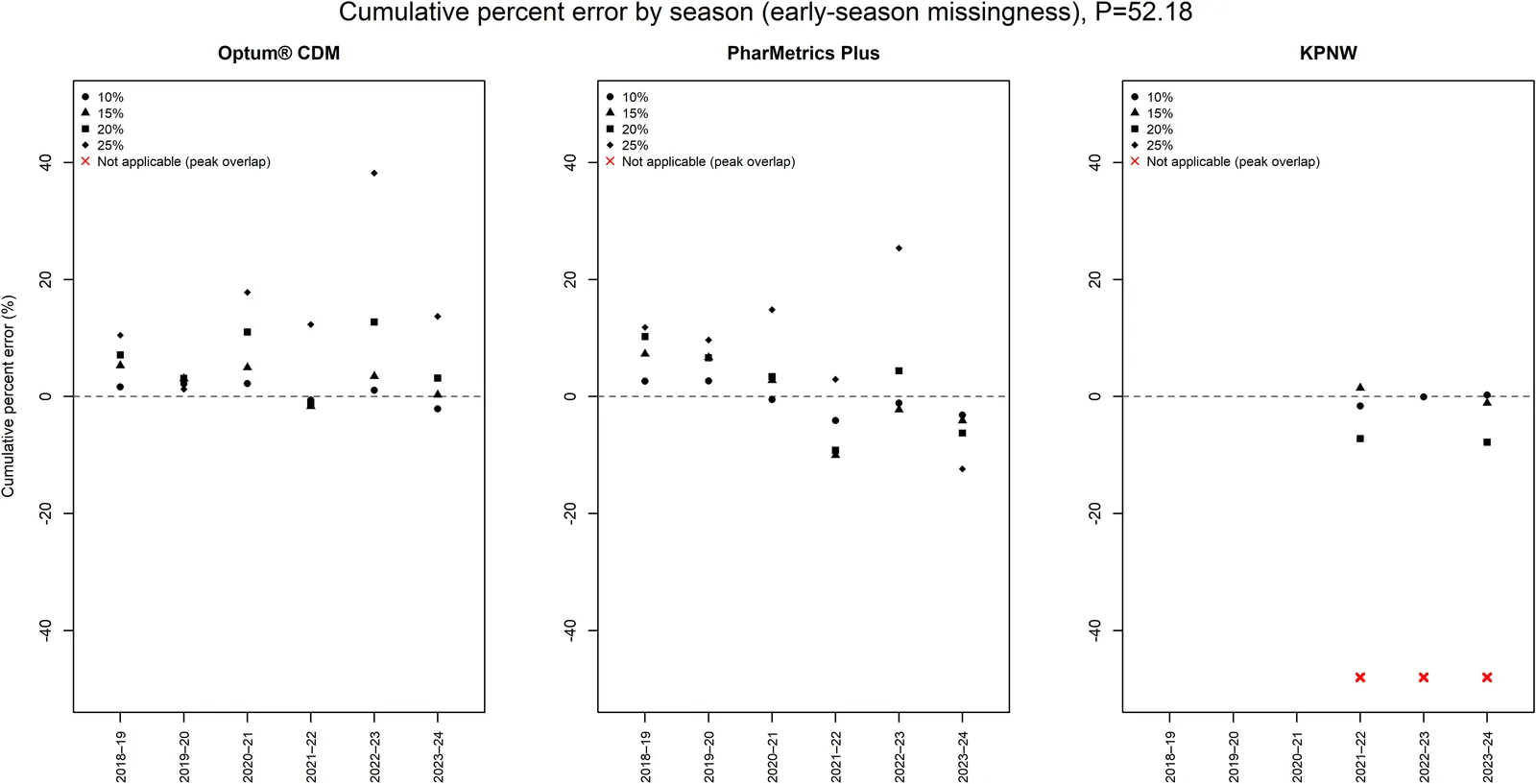
Cumulative percent error of reconstructed RSV seasonal incidence for early-season missingness scenarios, by data source, season, and missingness level. Points show cumulative percent error of reconstructed seasonal RSV clinical incidence (per 100,000) relative to the observed season total for each season and missingness level. Scenarios classified as not applicable because the omitted block overlapped the seasonal peak are indicated with a red “×”. RSV: Respiratory syncytial virus.

**Fig 4:**
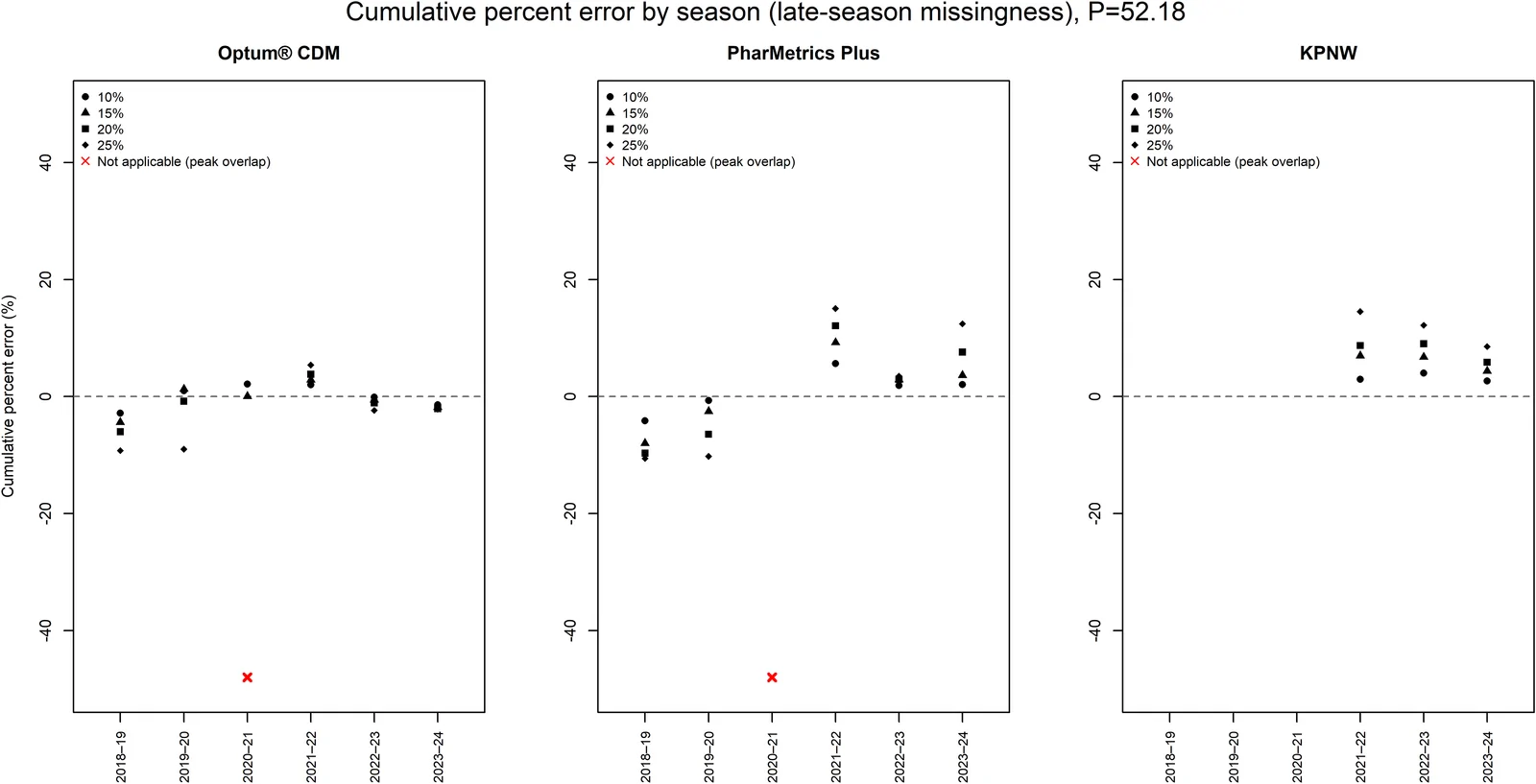
Cumulative percent error of reconstructed RSV seasonal incidence for late-season missingness scenarios, by data source, season, and missingness level. Points show cumulative percent error of reconstructed seasonal RSV clinical incidence (per 100,000) relative to the observed season total for each season and missingness level. Scenarios classified as not applicable because the omitted block overlapped the seasonal peak are indicated with a red “×”. RSV: Respiratory syncytial virus.

Season-by-season cumulative percent errors by source and missingness level are shown in Figs 3 and 4, with peak-overlap scenarios explicitly marked as “not applicable”. Weekly and cumulative exemplar reconstructions for early- and late-season missingness are shown in Figs 1 and 2, including both the “do nothing” incomplete baseline and dependence-aware predictive uncertainty. Detailed season-level results and additional diagnostics (including season-specific coefficient stability, overlap-alignment diagnostics, sensitivity to period specification, and bootstrap confidence intervals for median performance metrics) are provided in the Supporting Information (S2, S3, S4 and S7 Tables; Figs 3 and 4; S2 File).

Season-by-season stability analyses showed that the surveillance-to-RWD association varied across years (S5 Table), supporting the use of season-specific model fits rather than assuming a constant mapping across seasons. Overlap correlations were generally high in applicable scenarios (≥ 0.7), and scenarios without sufficient overlap were designated not applicable. Bootstrap 95% confidence intervals for median error metrics (percent error, signed difference, log-ratio error) are provided in the Supplement (S2 Table).

Using MBB prediction intervals to account for serial correlation, weekly calibration showed a clear side- and missingness‑pattern: late‑season coverage was consistently high across sources (medians near 1.0 for most bands), whereas early‑season coverage declined as the omitted block grew, with representative medians of about 0.52 for Optum^®^ CDM at 25% early and about 0.44 for PharMetrics Plus at 25% early. Despite lower early coverage, cumulative reconstruction errors remained small in most attempted scenarios (Table 1). Negative weekly predictions were uncommon and occurred primarily in late-season tail scenarios. For example, no early-season scenarios produced negative predictions, whereas Optum late 25% had negative values in 3/5 applicable season-scenarios (median proportion of held-out weeks truncated 0.25; S7 Table, S2 File). Excluding the truncated 2023–24 season did not materially change conclusions: weekly summaries were similar (Optum^®^ CDM showed slightly lower RMSE with comparable applicability; PharMetrics Plus had small early RMSE gains but lower late coverage; KPNW saw modest RMSE increases and slight late‑coverage declines), and early/late cumulative percent‑error medians shifted only modestly by band, confirming headline findings are robust to excluding 2023–24 (S8, S9 and S10 Tables). Across early ≈20% scenarios, non‑negative GLM and OLS produced similar reconstructed season totals (small percent‑error differences in Optum^®^ CDM and PharMetrics Plus), with larger gaps confined to KPNW and atypical/truncated seasons (S11 Table). For each scenario, we report the incomplete (no‑imputation) cumulative total alongside the reconstructed and observed totals, showing the practical gain from our method over the doing nothing (S12 Table).

### hMPV test-positivity proportions

**Fig 5:**
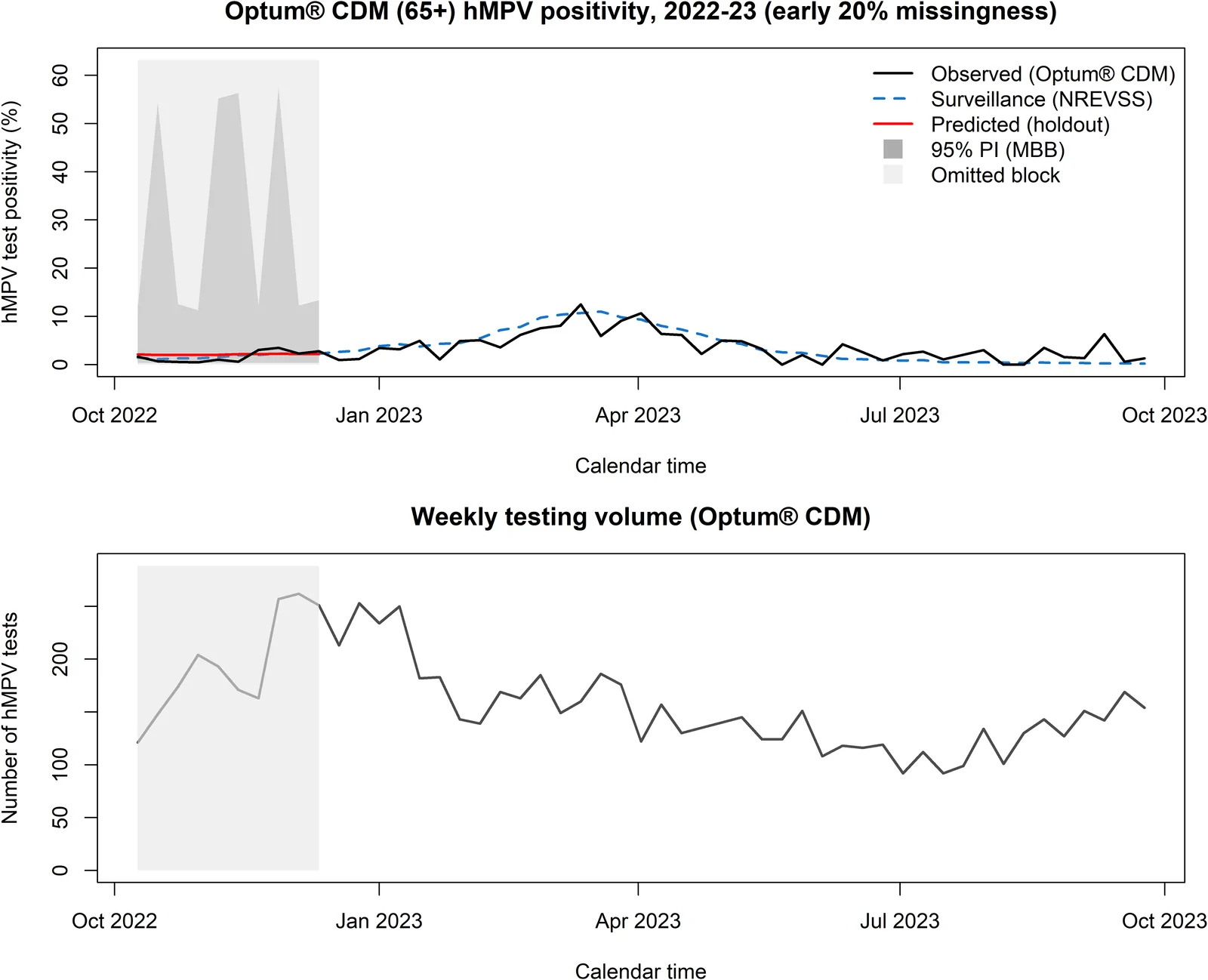
Illustrative reconstruction of weekly hMPV test positivity (%) in Optum^®^ CDM (adults ≥65 years) under a 20% early season missingness scenario (season 2022–23). Observed Optum^®^ CDM positivity is shown in black; the omitted block is shaded. Predicted positivity for omitted weeks is shown in red with 95% prediction intervals generated using a moving block bootstrap. The dashed blue line shows NREVSS hMPV test positivity used as the surveillance-aligned covariate. The lower panel shows weekly hMPV testing volume in Optum^®^ CDM. hMPV: Human metapneumovirus.

**Fig 6:**
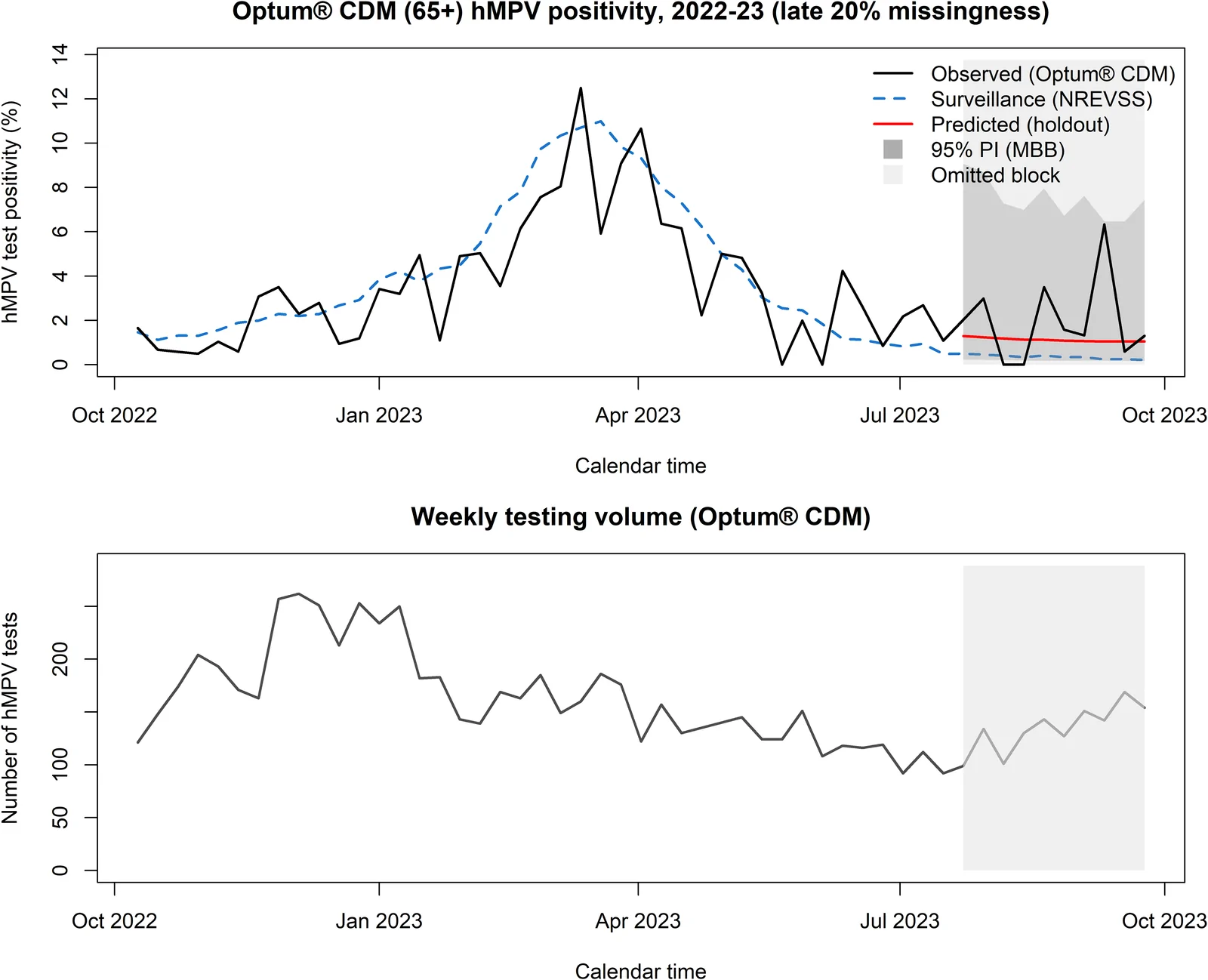
Illustrative reconstruction of weekly hMPV test positivity (%) in Optum^®^ CDM (adults ≥65 years) under a 20% late season missingness scenario (season 2022–23). Observed Optum^®^ CDM positivity is shown in black; the omitted block is shaded. Predicted positivity for omitted weeks is shown in red with 95% prediction intervals generated using a moving block bootstrap. The dashed blue line shows NREVSS hMPV test positivity used as the surveillance-aligned covariate. The lower panel shows weekly hMPV testing volume in Optum^®^ CDM. hMPV: Human metapneumovirus.

The hMPV analyses were conducted as an illustrative, exploratory extension of the approach to a bounded, denominator-based outcome, using Optum^®^ CDM (adults ≥65 years) for the 2022–23 and 2023–24 seasons and NREVSS hMPV test positivity as the surveillance-aligned covariate. Across early- and late-season missingness scenarios, weekly hMPV positivity predictions showed RMSE on the order of ~1–2 percentage points (Table 4). An exemplar reconstruction is shown in Fig 5 and Fig 6 (also in S1 and S2 Figs), including the omitted block, predicted values with 95% PI, and weekly testing volume to contextualize variability in precision.

**Table 4:** Illustrative weekly prediction performance for hMPV test positivity in Optum^®^ CDM (adults ≥65 years), seasons 2022–23 and 2023–24. Values are median (IQR) across seasons (n = 2). RMSE is reported in percentage points. Prediction intervals are pointwise 95% intervals generated using a moving block bootstrap.

Missingness location (early/late)	Missingness level (% of weeks omitted)	Seasons evaluated, n	RMSE (percentage points), median (IQR)	Empirical 95% PI coverage, median (IQR)	95% PI width (percentage points), median (IQR)
Early	10%	2	1.15 (0.17)	1.000 (0.000)	11.82 (1.59)
Early	15%	2	1.03 (0.24)	1.000 (0.000)	12.51 (1.56)
Early	20%	2	0.91 (0.22)	1.000 (0.000)	17.50 (2.95)
Early	25%	2	0.97 (0.34)	1.000 (0.000)	30.88 (16.12)
Late	10%	2	1.91 (0.40)	0.900 (0.100)	8.45 (1.29)
Late	15%	2	1.69 (0.40)	0.750 (0.125)	9.56 (2.68)
Late	20%	2	1.62 (0.40)	0.850 (0.050)	10.74 (3.27)
Late	25%	2	1.62 (0.23)	0.885 (0.038)	13.94 (5.92)

95% PI: 95% Prediction intervals; hMPV: Human metapneumovirus; IQR: Interquartile range; nRMSE: Normalized root mean squared error; RMSE: Root mean squared error. nRMSE was not reported for hMPV because the outcome is test positivity (%), which is already on a common bounded scale across seasons; RMSE in percentage points provides a directly interpretable error measure without additional normalization.

## Discussion

Our findings highlight the value of consistent burden studies and surveillance, and the need to maximize partial data. Across six RSV seasons, epidemic dynamics varied by attack rate, timing, and peak magnitude yet were broadly consistent across RWD sources. Pandemic-related disruptions were evident (suppressed 2020–21 activity and atypical 2021–22 and 2022–23 timing), with 2023–24 resembling pre‑pandemic patterns. For hMPV, pronounced week‑to‑week variability likely reflects inconsistent testing.

Predicted weekly values generally aligned with observations across seasons and data sources in applicable scenarios, i.e., when missingness was confined to contiguous early- or late-season weeks and did not remove peak activity. Late-season corrections tended to show smaller cumulative percent error than early-season corrections. Mechanistically, this follows from the model structure: when late-season weeks are missing, the observed portion of the season includes the rise and peak, which provides the strongest information for estimating the seasonal phase (harmonic terms) and the surveillance-to-study scaling. Predictions for the late tail are therefore primarily a short extrapolation from a well-identified peak. By contrast, early-season missingness requires reconstructing the pre-peak rise using only mid/late-season observations, making predictions more sensitive to shifts in season onset timing and early growth rate across years, and more dependent on extrapolation.

High weekly RMSE (e.g., Optum^®^ CDM with 15% early-season missingness in 2022–23; Pharmetrics Plus with 10–15% in 2023–24) still yielded low cumulative-incidence error, indicating that accurate season totals are achievable even when week-level predictions are imperfect. Early-season under-coverage is expected: with shorter training and steep early ramps, residual variance is higher and less stationary, so block-bootstrapped weekly PIs can under-cover. Our estimand is the seasonal total, and aggregation dampens week-level misspecification, yielding robust cumulative accuracy. Operationally, we exclude peak-overlap via a pre-specified screen and treat weekly PIs as diagnostics, not primary inference.

The hMPV results should be interpreted as illustrative and exploratory, given the limited evaluation (two seasons, one RWD source) and the use of test positivity rather than incidence. Positivity is influenced by testing practices and test volume and therefore reflects a different measurement process than incidence. Despite these constraints, the framework demonstrates how surveillance-aligned signals can be used to reconstruct contiguous early- or late-season gaps for bounded outcomes. Broader assessment across additional seasons, data sources, and surveillance strata would be needed to draw strong conclusions about generalizability for hMPV. If test-positivity reflects national hMPV dynamics, future work could map surveillance positivity to study-specific incidence estimates.

The three RWD sources were selected to also represent varying degrees of diagnostic certainty, ranging from only using diagnostic codes to ascertain RSV/hMPV status to using a combination of both diagnostic codes and laboratory tests. While a direct association between model performance and RSV/hMPV case ascertainment method was not found, a steady increase in the cumulative incidence of RSV with every season (except for the 2020–21 season) across RWD sources was noted. In addition to the evolving epidemiology of the virus, this also likely reflects an improvement in testing practices for RSV over the years across the RWD sources used. While the overall trend of the estimated incidence in our analysis was similar the surveillance data, caution when interpreting absolute values of incidence reported in this paper is advised, as reverse-transcriptase polymerase chain reaction testing for RSV/hMPV is not offered as a standard of care for patients presenting with acute respiratory infection (ARI) in any of the RWD sources analyzed. RSV-Net measures hospitalization incidence, whereas RWD outcomes reflect coded clinical events whose detection depends on testing and coding practices that can vary by age (e.g., differing thresholds for RSV testing, care-seeking, and hospitalization). Because of these differences, our model reconstructs the RWD clinical event time series using surveillance timing, it does not estimate population infection incidence. Claims are therefore limited to improving comparability and completeness of study‑specific clinical incidence totals. A more accurate ascertainment of infection status through routine testing of patients presenting with ARI, as would be done in a prospective burden of disease study, will likely further improve model performance.

Our method and its intended use case is for retrospective correction of partial‑season series. We do not assess nowcasting or prospective forecasting. While our method offers valuable solutions for estimating incidence incomplete time series data, it is important to recognize its limitations and appropriate use cases. Our method assumes that the study time series and surveillance share broadly aligned seasonal dynamics over the observed overlap. To support practical use of this method when only partial local data are available, we recommend several simple applicability checks using the observed overlap between the local series and the surveillance signal. First, visually compare trajectories over the overlapping weeks to assess whether the rise, peak timing, and decline are broadly aligned. Second, quantify alignment by estimating the correlation between the observed local series and surveillance during the overlap, and by fitting the proposed regression model on the overlap to confirm a positive, stable association. As a pragmatic heuristic, correlations of approximately 0.7 or higher suggest reasonable temporal alignment, whereas substantially lower correlations indicate a higher risk of divergence and unstable reconstructions. Third, assess whether peak activity is included in the observed segment by screening using surveillance data only: before attempting correction, identify the surveillance peak week for the season (t_peak = arg max of the aligned RSV‑Net series for the relevant geography/age group). For an early gap of n weeks (weeks 1…n) or a late gap (weeks T − n + 1…T), classify the scenario as not applicable if the omitted block includes t_peak. Missing peak weeks is a primary failure mode and we treat such scenarios as not applicable. Finally, consider the likely proportion of seasonal burden in the missing block (which can differ substantially even for the same proportion of weeks missing) and use geographically appropriate surveillance where available (e.g., state/regional surveillance for single-site studies rather than national trends). In our season-by-season stability analysis, coefficient magnitudes and model fit varied across seasons, consistent with pandemic disruption and evolving testing practices. We recommend applying our method only when the omitted block does not intersect the surveillance peak and when the observed overlap with surveillance shows reasonable alignment (e.g., positive slope, correlation ≥0.6–0.7). Otherwise, users should refrain from correction or consider alternate approaches. It may also be prudent to perform a validation exercise against previous season data within the planned data source, if available. Our method is optimally suited for correcting seasonal epidemic data missing a few weeks either at the start or the end of the incidence curve. The surveillance series used here should be viewed as an imperfect temporal anchor rather than a gold standard measure of true infection incidence. Also, our evaluation was conducted using U.S.-based surveillance systems and U.S. real‑world data sources. Although the framework is general, applying it in other countries or health systems may require careful selection of an appropriate surveillance signal and additional validation given differences in case definitions, care‑seeking, and seasonal timing.

In downstream analyses, uncertainty from the imputed weeks should be propagated rather than treating reconstructed values as fixed. One practical approach is to treat the imputation procedure as generating multiple plausible completed time series (e.g., via bootstrap-based predictive draws), rerun the downstream analysis for each completed series (seasonal totals, group comparisons, or burden estimates), and summarize the resulting distribution of estimates (e.g., median and 95% interval). Alternatively, if results are combined across studies (e.g., meta-analysis), investigators can apply standard multiple-imputation combining rules by pooling point estimates and within-imputation standard errors across imputed datasets. When only seasonal cumulative incidence is required, uncertainty can be propagated by generating bootstrap draws of the season total directly and carrying those forward to subsequent analyses. For meta-analysis, compute the study-level effect estimate within each imputed dataset and then pool across imputations (or bootstrap draws) to obtain an overall estimate and interval that reflects both sampling and imputation uncertainty.

Our method is a novel application of established time series regression methodology and addresses two primary challenges in epidemiological research of seasonal respiratory viruses: incomplete data in prospective burden of disease studies and exclusion of studies with incomplete data from systematic reviews. This approach may be extended to other seasonal respiratory pathogens when an aligned surveillance signal exists and missingness is confined to contiguous early/late-season weeks that do not remove peak activity. Prospective studies may miss parts of the season due to operational delays, unexpected viral timing (early-season gaps), or unplanned early termination (late-season gaps). Many are designed around the seasonality of one respiratory virus and may, therefore, not fully capture seasonality of other. These scenarios inevitably lead to underestimation of cumulative incidence, potentially skewing our understanding of disease burden. Systematic reviews frequently encounter similar challenges when synthesizing data from various sources. Current practices often necessitate the separation of studies presenting seasonal estimates from those offering annual data [13–18]. In some cases, studies with incomplete seasonal data are entirely excluded from the review process, resulting in a loss of valuable information [7,8,19]. Our approach does not aim to supplant more sophisticated frameworks (e.g., multiple imputation, likelihood/EM, or state-space models), which can be preferable when strong parametric assumptions hold, missingness mechanisms are well characterized, or high-fidelity weekly reconstruction is the goal [20–23]. However, such methods require specialized software, heavier computation, and careful specification, and they are seldom used in routine prospective studies or systematic reviews, where partial-season studies are often excluded. We offer a pragmatic alternative: when missingness is confined to contiguous early- or late-season weeks, peaks are not entirely missing, and local dynamics align with surveillance seasonality, simple linear regression with harmonic terms can yield cumulative incidence estimates with low relative error despite modest pointwise RMSE near peaks [24]. This can improve cumulative incidence accuracy in prospective studies, enable inclusion of previously excluded partial-season data in reviews, and enhance comparability across different reporting periods. Ultimately, our method aims to maximize the utility of available data, leading to more comprehensive and accurate assessments of respiratory virus burden.

## Supporting information

S1 Fig Illustrative reconstruction of weekly hMPV test positivity (%) in Optum®CDM (adults ≥65 years) under a 20% early season missingness scenario (season2023–24). (PDF)

S2 Fig Illustrative reconstruction of weekly hMPV test positivity (%) in Optum®CDM (adults ≥65 years) under a 20% late season missingness scenario (season 2023–24). (PDF)

S1 Table Diagnostic and positive laboratory test codes used to determine RSV and hMPV infection status in Optum® CDM, KPNW, and PharMetrics Plus databases. (PDF)

S2 Table Bootstrap 95% CIs for median metrics. (PDF)

S3 Table Applicability/peak-overlap rates. (PDF)

S4 Table Period sensitivity P=52 vs. P=52.18, median (IQR) across paired scenarios. (PDF)

S5 Table Season‑by‑season stability (P = 52.18). (PDF)

S6 Table Correlation diagnostics vs error. (PDF)

S7 Table Scenario-level results for all sources, seasons, and missingness scenarios (CSV). (PDF)

S8 Table Weekly reconstruction performance and applicability for RSV clinical incidence (P = 52.18), sensitivity analysis excluding the 2023-24 season. (PDF)

S9 Table Seasonal cumulative reconstruction accuracy for RSV clinical incidence under early-season missingness (P = 52.18), sensitivity analysis excluding the 2023–24 season. (PDF)

S10 Table Seasonal cumulative reconstruction accuracy for RSV clinical incidence under late-season missingness (P = 52.18), sensitivity analysis excluding the 2023-24 season. (PDF)

S11 Table Sensitivity analysis using non‑negative link (quasi‑Poisson/gamma‑log) for early ≈20% gaps: Comparison of Ordinary Least Squares and Gamma Generalized Linear Model reconstructed cumulative RSV incidence, percent errors, and deltas by source and season. (PDF)

S12 Table Incomplete vs reconstructed vs observed cumulative RSV incidence by scenario (source× season × side × missingness). (PDF)

S1 File Annotated R code demonstrating the RSV and hMPV imputation workflows on simulated data. All author-generated code used for data preprocessing, model fitting, prediction/imputation, and figure/table generation have been provided. Because the underlying real-world datasets are proprietary, the repository also includes a complete simulated-data example that reproduces the full analysis workflow.
(ZIP)

S2 File Scenario-level results for all sources, seasons, and missingness scenarios. A expanded version of the data contained in the S7 table.
(CSV)
