# Supplementary material for: Estimating cumulative incidence from partially missing time series of respiratory viral infections
Source: PLoS One. 2026 Sep 15;21(9):e0353681. doi: 10.1371/journal.pone.0353681 (PMC13577586; doi:10.1371/journal.pone.0353681)

S2 Figure. Illustrative reconstruction of weekly hMPV test positivity (%) in Optum® CDM (adults ≥65 years) under a 20% late season missingness scenario (season 2023–24)

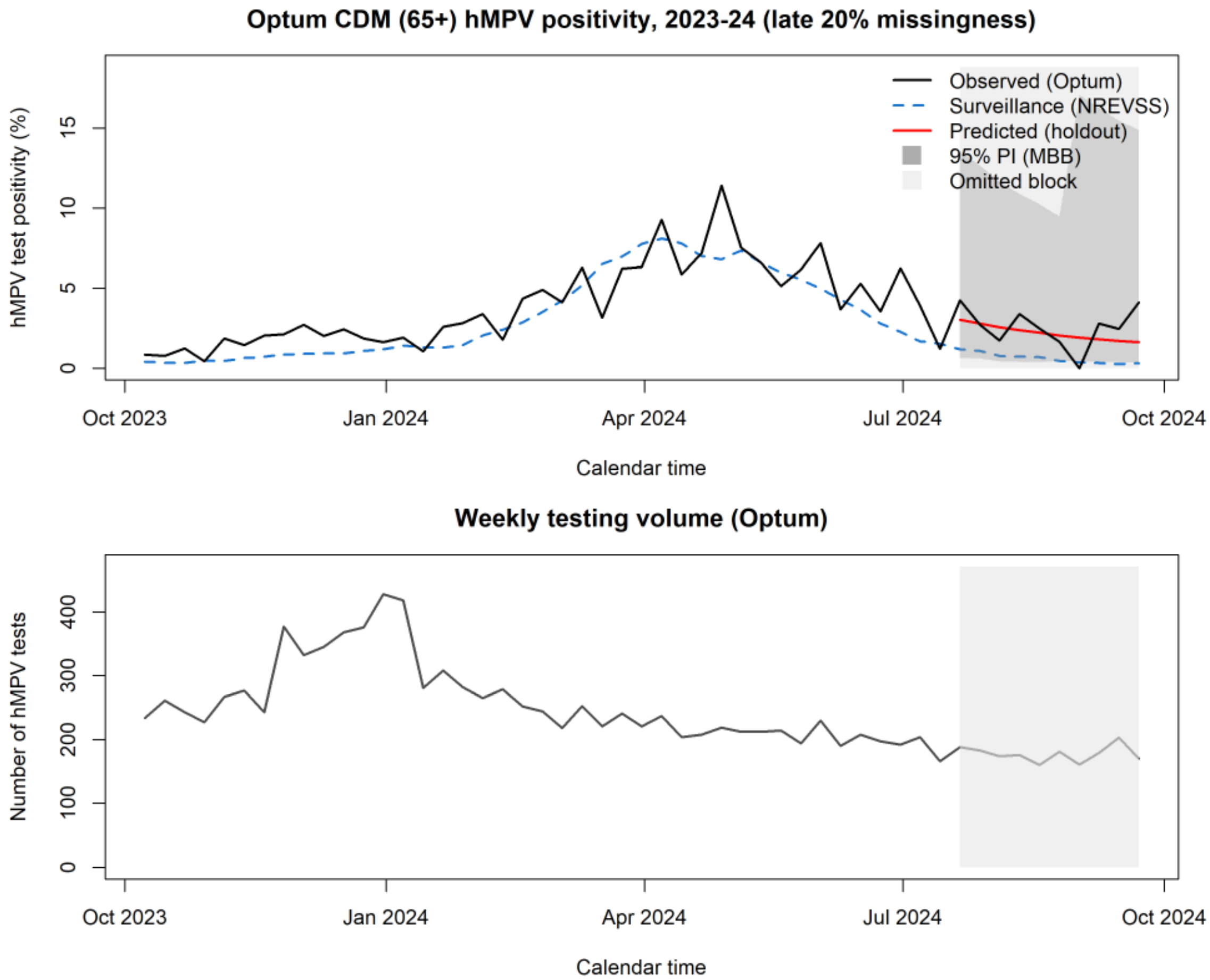

Supplement: Supplementary file 2 [file pone.0353681.s002.pdf]
