# Supplementary material for: Estimating cumulative incidence from partially missing time series of respiratory viral infections
Source: PLoS One. 2026 Sep 15;21(9):e0353681. doi: 10.1371/journal.pone.0353681 (PMC13577586; doi:10.1371/journal.pone.0353681)
Supplement: Supplementary file 3 [file pone.0353681.s003.pdf]

S1 Table. Diagnostic and positive laboratory test codes used to determine RSV and hMPV infection status in Optum® CDM, KPNW, and PharMetrics Plus databases.

**RSV diagnostic codes (used across all real-word data sources)**		
**Code**	**Type**	**Description**
79.6	ICD-9	RSV
466.11	ICD-9	Acute bronchiolitis due to RSV
480.1	ICD-9	Pneumonia due to respiratory syncytial virus
J12.1	ICD-10	RSV pneumonia
J20.5	ICD-10	Acute bronchitis due to RSV
J21.0	ICD-10	Acute bronchiolitis due to RSV
B97.4	ICD-10	RSV as the cause of diseases classified elsewhere
**RSV laboratory test codes (used for Optum® CDM and KPNW)**		
**Code**	**Type**	**Description**
14129-1	LOINC	RSV [Presence] in Nose by Organism specific culture
17517-4	LOINC	RSV IgG antibody [Titer] in Cerebral spinal fluid
17518-2	LOINC	RSV IgG antibody [Titer] in Serum
17519-0	LOINC	RSV IgM antibody [Titer] in Cerebral spinal fluid
17520-8	LOINC	RSV identified in Unspecified specimen by Organism specific culture
22466-7	LOINC	RSV antibody [Titer] in Serum
22467-5	LOINC	RSV IgM antibody [Titer] in Serum
24224-8	LOINC	RSV antibody [Titer] in Serum by Complement fixation --1st specimen
24225-5	LOINC	RSV antibody [Titer] in Serum by Complement fixation --2nd specimen
24298-2	LOINC	RSV antibody [Titer] in Serum --1st specimen
24299-0	LOINC	RSV antibody [Titer] in Serum --2nd specimen
30075-6	LOINC	RSV A RNA [Presence] in Unspecified specimen by NAA with probe detection
30076-4	LOINC	RSV B RNA [Presence] in Unspecified specimen by NAA with probe detection
30147-3	LOINC	RSV antibody [Presence] in Serum --1st specimen
30148-1	LOINC	RSV antibody [Presence] in Serum --2nd specimen
31583-8	LOINC	RSV IgG antibody [Units/volume] in Cerebral spinal fluid
31949-1	LOINC	RSV antigen [Presence] in Throat
31950-9	LOINC	RSV antigen [Presence] in Unspecified specimen
32040-8	LOINC	RSV antigen [Presence] in Nose by Immunofluorescence
33045-6	LOINC	RSV antigen [Presence] in Nose
33382-3	LOINC	RSV antibody [Titer] in Cerebral spinal fluid
33390-6	LOINC	RSV antibody [Presence] in Serum
40987-0	LOINC	RSV RNA [Identifier] in Unspecified specimen by NAA with probe detection
40988-8	LOINC	RSV RNA [Presence] in Unspecified specimen by NAA with probe detection
40989-6	LOINC	RSV IgG antibody [Presence] in Serum by Immunoassay
**hMPV diagnostic codes (used for Optum® CDM)**		
**Code**	**Type**	**Description**
B97.81	ICD-10	hMPV as the cause of diseases classified elsewhere
J12.3	ICD-10	hMPV pneumonia
J21.1	ICD-10	Acute bronchiolitis due to hMPV
**hMPV laboratory test codes (used for Optum® CDM)**		
**Code**	**Type**	**Description**
38917-1	LOINC	HMPV RNA [Presence] in Unspecified specimen by NAA with probe detection
40978-9	LOINC	HMPV RNA [Identifier] in Unspecified specimen by NAA with probe detection
40979-7	LOINC	HMPV antigen [Presence] in Unspecified specimen by Immunofluorescence
40980-5	LOINC	HMPV IgG antibody [Presence] in Serum by Immunoassay

41011-8	LOINC	HMPV IgG antibody [Presence] in Serum
41455-7	LOINC	Deprecated HMPV RNA [Identifier] in Unspecified specimen by Probe & target amplification method
53249-9	LOINC	HMPV RNA [#/volume] (viral load) in Unspecified specimen by NAA with probe detection
60266-4	LOINC	HMPV RNA [Presence] in Isolate by NAA with probe detection
60425-6	LOINC	HMPV antigen [Presence] in Unspecified specimen
67820-1	LOINC	HMPV A RNA [Presence] in Unspecified specimen by NAA with probe detection
67821-9	LOINC	HMPV B RNA [Presence] in Unspecified specimen by NAA with probe detection
77024-8	LOINC	HMPV RNA [Presence] in Nasopharynx by NAA with probe detection
80587-9	LOINC	HMPV A+B L+N genes [Presence] in Nasopharynx by NAA with probe detection
82165-2	LOINC	HMPV RNA [Presence] in Nasopharynx by NAA with non-probe detection
88222-5	LOINC	HMPV antigen [Presence] in Nasopharynx by Immunofluorescence
88532-7	LOINC	HMPV identified in Cornea or Conjunctiva by Organism specific culture
88534-3	LOINC	HMPV RNA [Presence] in Cornea or Conjunctiva by NAA with probe detection
89651-4	LOINC	HMPV RNA [Presence] in Lower respiratory specimen by NAA with probe detection
89652-2	LOINC	HMPV identified in Lower respiratory specimen by Organism specific culture
91778-1	LOINC	HMPV identified in Upper respiratory specimen by Organism specific culture
91809-4	LOINC	HMPV RNA [Presence] in Upper respiratory specimen by NAA with probe detection
91810-2	LOINC	HMPV antigen [Presence] in Upper respiratory specimen by Immunofluorescence
91831-8	LOINC	HMPV antigen [Presence] in Lower respiratory specimen by Immunofluorescence
92134-6	LOINC	HMPV RNA [Presence] in Respiratory specimen by NAA with probe detection
92978-6	LOINC	HMPV RNA [Presence] in Lower respiratory specimen by NAA with non-probe detection

Definition of RSV and hMPV: At least one diagnostic code from physicians or a positive result from a laboratory test (Optum® CDM only) on the analysis week

hMPV: Human metapneumovirus; RSV: Respiratory syncytial virus.
