# Supplementary material for: Estimating cumulative incidence from partially missing time series of respiratory viral infections
Source: PLoS One. 2026 Sep 15;21(9):e0353681. doi: 10.1371/journal.pone.0353681 (PMC13577586; doi:10.1371/journal.pone.0353681)
Supplement: Supplementary file 4 [file pone.0353681.s004.pdf]

S2 Table. Bootstrap 95% CIs for median metrics

**Data source**	**Missingness location** (early/late)	**Missingness level** (% of weeks omitted)	**Performance metric**	**Seasons resampled,** n	**Median [95% CI]**
KPNW	early	10%	rmse_holdout	6	0.770 [0.479, 1.192]
KPNW	early	15%	rmse_holdout	6	0.770 [0.479, 1.192]
KPNW	early	20%	rmse_holdout	6	0.770 [0.479, 1.192]
KPNW	early	25%	rmse_holdout	6	0.770 [0.479, 1.192]
KPNW	late	10%	rmse_holdout	6	0.770 [0.479, 1.192]
KPNW	late	15%	rmse_holdout	6	0.770 [0.479, 1.192]
KPNW	late	20%	rmse_holdout	6	0.770 [0.479, 1.192]
KPNW	late	25%	rmse_holdout	6	0.770 [0.479, 1.192]
Optum	early	10%	rmse_holdout	6	0.770 [0.479, 1.192]
Optum	early	15%	rmse_holdout	6	0.770 [0.479, 1.192]
Optum	early	20%	rmse_holdout	6	0.770 [0.479, 1.192]
Optum	early	25%	rmse_holdout	6	0.770 [0.479, 1.192]
Optum	late	10%	rmse_holdout	6	0.770 [0.479, 1.192]
Optum	late	15%	rmse_holdout	6	0.770 [0.479, 1.192]
Optum	late	20%	rmse_holdout	6	0.770 [0.479, 1.192]
Optum	late	25%	rmse_holdout	6	0.770 [0.479, 1.192]
PharMetrics	early	10%	rmse_holdout	6	0.770 [0.479, 1.192]
PharMetrics	early	15%	rmse_holdout	6	0.770 [0.479, 1.192]
PharMetrics	early	20%	rmse_holdout	6	0.770 [0.479, 1.192]
PharMetrics	early	25%	rmse_holdout	6	0.770 [0.479, 1.192]

PharMetrics	late	10%	rmse_holdout	6	0.770 [0.479, 1.192]
PharMetrics	late	15%	rmse_holdout	6	0.770 [0.479, 1.192]
PharMetrics	late	20%	rmse_holdout	6	0.770 [0.479, 1.192]
PharMetrics	late	25%	rmse_holdout	6	0.770 [0.479, 1.192]
KPNW	early	10%	nrmse_holdout	6	0.331 [0.222, 0.466]
KPNW	early	15%	nrmse_holdout	6	0.331 [0.222, 0.466]
KPNW	early	20%	nrmse_holdout	6	0.331 [0.222, 0.466]
KPNW	early	25%	nrmse_holdout	6	0.331 [0.222, 0.466]
KPNW	late	10%	nrmse_holdout	6	0.331 [0.222, 0.466]
KPNW	late	15%	nrmse_holdout	6	0.331 [0.222, 0.466]
KPNW	late	20%	nrmse_holdout	6	0.331 [0.222, 0.466]
KPNW	late	25%	nrmse_holdout	6	0.331 [0.222, 0.466]
Optum	early	10%	nrmse_holdout	6	0.331 [0.222, 0.466]
Optum	early	15%	nrmse_holdout	6	0.331 [0.222, 0.466]
Optum	early	20%	nrmse_holdout	6	0.331 [0.222, 0.466]
Optum	early	25%	nrmse_holdout	6	0.331 [0.222, 0.466]
Optum	late	10%	nrmse_holdout	6	0.331 [0.222, 0.466]
Optum	late	15%	nrmse_holdout	6	0.331 [0.222, 0.466]
Optum	late	20%	nrmse_holdout	6	0.331 [0.222, 0.466]
Optum	late	25%	nrmse_holdout	6	0.331 [0.222, 0.466]
PharMetrics	early	10%	nrmse_holdout	6	0.331 [0.222, 0.466]
PharMetrics	early	15%	nrmse_holdout	6	0.331 [0.222, 0.466]

PharMetrics	early	20%	nrmse_holdout	6	0.331 [0.222, 0.466]
PharMetrics	early	25%	nrmse_holdout	6	0.331 [0.222, 0.466]
PharMetrics	late	10%	nrmse_holdout	6	0.331 [0.222, 0.466]
PharMetrics	late	15%	nrmse_holdout	6	0.331 [0.222, 0.466]
PharMetrics	late	20%	nrmse_holdout	6	0.331 [0.222, 0.466]
PharMetrics	late	25%	nrmse_holdout	6	0.331 [0.222, 0.466]
KPNW	early	10%	pi_coverage95	6	0.858 [0.759, 1.000]
KPNW	early	15%	pi_coverage95	6	0.858 [0.759, 1.000]
KPNW	early	20%	pi_coverage95	6	0.858 [0.759, 1.000]
KPNW	early	25%	pi_coverage95	6	0.858 [0.759, 1.000]
KPNW	late	10%	pi_coverage95	6	0.858 [0.759, 1.000]
KPNW	late	15%	pi_coverage95	6	0.858 [0.759, 1.000]
KPNW	late	20%	pi_coverage95	6	0.858 [0.759, 1.000]
KPNW	late	25%	pi_coverage95	6	0.858 [0.759, 1.000]
Optum	early	10%	pi_coverage95	6	0.858 [0.759, 1.000]
Optum	early	15%	pi_coverage95	6	0.858 [0.759, 1.000]
Optum	early	20%	pi_coverage95	6	0.858 [0.759, 1.000]
Optum	early	25%	pi_coverage95	6	0.858 [0.759, 1.000]
Optum	late	10%	pi_coverage95	6	0.858 [0.759, 1.000]
Optum	late	15%	pi_coverage95	6	0.858 [0.759, 1.000]
Optum	late	20%	pi_coverage95	6	0.858 [0.759, 1.000]
Optum	late	25%	pi_coverage95	6	0.858 [0.759, 1.000]

PharMetrics	early	10%	pi_coverage95	6	0.858 [0.759, 1.000]
PharMetrics	early	15%	pi_coverage95	6	0.858 [0.759, 1.000]
PharMetrics	early	20%	pi_coverage95	6	0.858 [0.759, 1.000]
PharMetrics	early	25%	pi_coverage95	6	0.858 [0.759, 1.000]
PharMetrics	late	10%	pi_coverage95	6	0.858 [0.759, 1.000]
PharMetrics	late	15%	pi_coverage95	6	0.858 [0.759, 1.000]
PharMetrics	late	20%	pi_coverage95	6	0.858 [0.759, 1.000]
PharMetrics	late	25%	pi_coverage95	6	0.858 [0.759, 1.000]
KPNW	early	10%	cum_percent_error	6	2.11 [-0.18, 3.01]
KPNW	early	15%	cum_percent_error	6	2.11 [-0.18, 3.01]
KPNW	early	20%	cum_percent_error	6	2.11 [-0.18, 3.01]
KPNW	early	25%	cum_percent_error	6	2.11 [-0.18, 3.01]
KPNW	late	10%	cum_percent_error	6	2.11 [-0.18, 3.01]
KPNW	late	15%	cum_percent_error	6	2.11 [-0.18, 3.01]
KPNW	late	20%	cum_percent_error	6	2.11 [-0.18, 3.01]
KPNW	late	25%	cum_percent_error	6	2.11 [-0.18, 3.01]
Optum	early	10%	cum_percent_error	6	2.11 [-0.18, 3.01]
Optum	early	15%	cum_percent_error	6	2.11 [-0.18, 3.01]
Optum	early	20%	cum_percent_error	6	2.11 [-0.18, 3.01]
Optum	early	25%	cum_percent_error	6	2.11 [-0.18, 3.01]
Optum	late	10%	cum_percent_error	6	2.11 [-0.18, 3.01]

Optum	late	15%	cum_percent_error	6	2.11 [-0.18, 3.01]
Optum	late	20%	cum_percent_error	6	2.11 [-0.18, 3.01]
Optum	late	25%	cum_percent_error	6	2.11 [-0.18, 3.01]
PharMetrics	early	10%	cum_percent_error	6	2.11 [-0.18, 3.01]
PharMetrics	early	15%	cum_percent_error	6	2.11 [-0.18, 3.01]
PharMetrics	early	20%	cum_percent_error	6	2.11 [-0.18, 3.01]
PharMetrics	early	25%	cum_percent_error	6	2.11 [-0.18, 3.01]
PharMetrics	late	10%	cum_percent_error	6	2.11 [-0.18, 3.01]
PharMetrics	late	15%	cum_percent_error	6	2.11 [-0.18, 3.01]
PharMetrics	late	20%	cum_percent_error	6	2.11 [-0.18, 3.01]
PharMetrics	late	25%	cum_percent_error	6	2.11 [-0.18, 3.01]
KPNW	early	10%	cum_abs_diff	6	1.18 [0.06, 3.64]
KPNW	early	15%	cum_abs_diff	6	1.18 [0.06, 3.64]
KPNW	early	20%	cum_abs_diff	6	1.18 [0.06, 3.64]
KPNW	early	25%	cum_abs_diff	6	1.18 [0.06, 3.64]
KPNW	late	10%	cum_abs_diff	6	1.18 [0.06, 3.64]
KPNW	late	15%	cum_abs_diff	6	1.18 [0.06, 3.64]
KPNW	late	20%	cum_abs_diff	6	1.18 [0.06, 3.64]
KPNW	late	25%	cum_abs_diff	6	1.18 [0.06, 3.64]
Optum	early	10%	cum_abs_diff	6	1.18 [0.06, 3.64]
Optum	early	15%	cum_abs_diff	6	1.18 [0.06, 3.64]

Optum	early	20%	cum_abs_diff	6	1.18 [0.06, 3.64]
Optum	early	25%	cum_abs_diff	6	1.18 [0.06, 3.64]
Optum	late	10%	cum_abs_diff	6	1.18 [0.06, 3.64]
Optum	late	15%	cum_abs_diff	6	1.18 [0.06, 3.64]
Optum	late	20%	cum_abs_diff	6	1.18 [0.06, 3.64]
Optum	late	25%	cum_abs_diff	6	1.18 [0.06, 3.64]
PharMetrics	early	10%	cum_abs_diff	6	1.18 [0.06, 3.64]
PharMetrics	early	15%	cum_abs_diff	6	1.18 [0.06, 3.64]
PharMetrics	early	20%	cum_abs_diff	6	1.18 [0.06, 3.64]
PharMetrics	early	25%	cum_abs_diff	6	1.18 [0.06, 3.64]
PharMetrics	late	10%	cum_abs_diff	6	1.18 [0.06, 3.64]
PharMetrics	late	15%	cum_abs_diff	6	1.18 [0.06, 3.64]
PharMetrics	late	20%	cum_abs_diff	6	1.18 [0.06, 3.64]
PharMetrics	late	25%	cum_abs_diff	6	1.18 [0.06, 3.64]
KPNW	early	10%	cum_log_ratio_error	6	0.021 [-0.002, 0.030]
KPNW	early	15%	cum_log_ratio_error	6	0.021 [-0.002, 0.030]
KPNW	early	20%	cum_log_ratio_error	6	0.021 [-0.002, 0.030]

KPNW	early	25%	cum_log_ratio_error	6	0.021 [-0.002, 0.030]
KPNW	late	10%	cum_log_ratio_error	6	0.021 [-0.002, 0.030]
KPNW	late	15%	cum_log_ratio_error	6	0.021 [-0.002, 0.030]
KPNW	late	20%	cum_log_ratio_error	6	0.021 [-0.002, 0.030]
KPNW	late	25%	cum_log_ratio_error	6	0.021 [-0.002, 0.030]
Optum	early	10%	cum_log_ratio_error	6	0.021 [-0.002, 0.030]
Optum	early	15%	cum_log_ratio_error	6	0.021 [-0.002, 0.030]
Optum	early	20%	cum_log_ratio_error	6	0.021 [-0.002, 0.030]
Optum	early	25%	cum_log_ratio_error	6	0.021 [-0.002, 0.030]
Optum	late	10%	cum_log_ratio_error	6	0.021 [-0.002, 0.030]
Optum	late	15%	cum_log_ratio_error	6	0.021 [-0.002, 0.030]
Optum	late	20%	cum_log_ratio_error	6	0.021 [-0.002, 0.030]
Optum	late	25%	cum_log_ratio_error	6	0.021 [-0.002, 0.030]
PharMetrics	early	10%	cum_log_ratio_error	6	0.021 [-0.002, 0.030]

PharMetrics	early	15%	cum_log_ratio_error	6	0.021 [-0.002, 0.030]
PharMetrics	early	20%	cum_log_ratio_error	6	0.021 [-0.002, 0.030]
PharMetrics	early	25%	cum_log_ratio_error	6	0.021 [-0.002, 0.030]
PharMetrics	late	10%	cum_log_ratio_error	6	0.021 [-0.002, 0.030]
PharMetrics	late	15%	cum_log_ratio_error	6	0.021 [-0.002, 0.030]
PharMetrics	late	20%	cum_log_ratio_error	6	0.021 [-0.002, 0.030]
PharMetrics	late	25%	cum_log_ratio_error	6	0.021 [-0.002, 0.030]

CI: Confidence interval.
