# Supplementary material for: Estimating cumulative incidence from partially missing time series of respiratory viral infections
Source: PLoS One. 2026 Sep 15;21(9):e0353681. doi: 10.1371/journal.pone.0353681 (PMC13577586; doi:10.1371/journal.pone.0353681)
Supplement: Supplementary file 5 [file pone.0353681.s005.pdf]

**S3 Table. Applicability/peak-overlap rates**

| **Data source** | **Missingness location** (early/late) | **Missingness level** (% of weeks omitted) | **Scenarios evaluated,** n | **Applicable scenarios,** n | **Applicability rate** | **Peak-overlap scenarios,** n | **Peak-overlap rate** |
|---|---|---|---|---|---|---|---|
| KPNW | early | 10% | 3 | 3 | 1 | 0 | 0 |
| KPNW | early | 15% | 3 | 2 | 0.667 | 1 | 0.333 |
| KPNW | early | 20% | 3 | 2 | 0.667 | 1 | 0.333 |
| KPNW | early | 25% | 3 | 0 | 0 | 3 | 1 |
| KPNW | late | 10% | 3 | 3 | 1 | 0 | 0 |
| KPNW | late | 15% | 3 | 3 | 1 | 0 | 0 |
| KPNW | late | 20% | 3 | 3 | 1 | 0 | 0 |
| KPNW | late | 25% | 3 | 3 | 1 | 0 | 0 |
| Optum | early | 10% | 6 | 6 | 1 | 0 | 0 |
| Optum | early | 15% | 6 | 6 | 1 | 0 | 0 |
| Optum | early | 20% | 6 | 6 | 1 | 0 | 0 |
| Optum | early | 25% | 6 | 6 | 1 | 0 | 0 |
| Optum | late | 10% | 6 | 6 | 1 | 0 | 0 |
| Optum | late | 15% | 6 | 6 | 1 | 0 | 0 |
| Optum | late | 20% | 6 | 5 | 0.833 | 1 | 0.167 |
| Optum | late | 25% | 6 | 5 | 0.833 | 1 | 0.167 |
| PharMetrics | early | 10% | 6 | 6 | 1 | 0 | 0 |
| PharMetrics | early | 15% | 6 | 6 | 1 | 0 | 0 |
| PharMetrics | early | 20% | 6 | 6 | 1 | 0 | 0 |
| PharMetrics | early | 25% | 6 | 6 | 1 | 0 | 0 |
| PharMetrics | late | 10% | 6 | 5 | 0.833 | 1 | 0.167 |
| PharMetrics | late | 15% | 6 | 5 | 0.833 | 1 | 0.167 |
| PharMetrics | late | 20% | 6 | 5 | 0.833 | 1 | 0.167 |
| PharMetrics | late | 25% | 6 | 5 | 0.833 | 1 | 0.167 |
