# Supplementary material for: Estimating cumulative incidence from partially missing time series of respiratory viral infections
Source: PLoS One. 2026 Sep 15;21(9):e0353681. doi: 10.1371/journal.pone.0353681 (PMC13577586; doi:10.1371/journal.pone.0353681)
Supplement: Supplementary file 6 [file pone.0353681.s006.pdf]

S4. Table: Period sensitivity P=52 vs. P=52.18, *median (IQR) across paired scenarios*

**Data source**	**Missingness location** (early/late)	**Missingness level** (% of weeks omitted)	**Paired scenarios**, n	**Δ Weekly RMSE**, median	**Δ Weekly RMSE**, IQR	**Δ Weekly nRMSE**, median	**Δ Weekly nRMSE**, IQR	**Δ 95% PI coverage**, median	**Δ 95% PI coverage**, IQR	**Δ Cumulative percent error (%)**, median	**Δ Cumulative percent error (%)**, IQR	**Δ Cumulative signed difference (per 100,000)**, median	**Δ Cumulative signed difference (per 100,000)**, IQR	**Δ Cumulative log-ratio error**, median	**Δ Cumulative log-ratio error**, IQR
KPNW	early	10%	3	0.000983	0.006138	0.000218	0.000144	0	0	-0.04554	0.013952	-0.06867	0.073244	-0.00046	0.0014
KPNW	early	15%	2	0.000831	0.001858	0.000121	0.000475	0	0	-0.05282	0.02577	-0.09096	0.027555	-0.00052	0.00251
KPNW	early	20%	2	0.001006	0.002743	0.000454	0.000839	-0.1	0	-0.01916	0.033343	-0.02297	0.056212	-0.00021	0.0036
KPNW	late	10%	3	0.007511	0.004783	0.000995	0.001626	0	0	0.01002	0.018658	0.039327	0.028707	9.63197806430616e-05	0.00181
KPNW	late	15%	3	0.011866	0.006404	0.001757	0.001995	0	0.0625	0.028978	0.032739	0.102334	0.054078	0.000271	0.00306
KPNW	late	20%	3	0.013084	0.00789	0.00209	0.002256	0	0	0.042868	0.045953	0.140619	0.082554	0.000393	0.00422
KPNW	late	25%	3	0.010137	0.00971	0.002271	0.002001	0	0	0.061062	0.051975	0.14119	0.13193	0.000544	0.00456
Optum	early	10%	6	-0.00049	0.002555	-0.00018	0.000987	0	0	-0.00172	0.012187	-0.00143	0.01305	-1.69E-05	0.0012

Optum	early	15%	6	-0.00112	0.001956	-0.00029	0.000823	0	0	-0.00622	0.017941	-0.00504	0.015965	-5.94E-05	0.000178
Optum	early	20%	6	0.000665	0.001392	0.000347	0.000573	0	0	0.004363	0.036157	0.001969	0.052856	3.80918166548398e-05	0.000333
Optum	early	25%	6	0.000409	0.004436	0.000439	0.001173	-0.03846	0.076923	0.013351	0.081446	0.007534	0.141305	0.000113	0.000601
Optum	late	10%	6	0.000463	0.001945	0.00036	0.001169	0	0.15	0.005538	0.011948	0.003621	0.010512	5.45311784905175e-05	0.000117
Optum	late	15%	6	-1.65E-05	0.002392	0.000169	0.001165	0	0	0.009393	0.02145	0.005827	0.020676	9.48657971621683e-05	0.000209
Optum	late	20%	5	0.000118	0.0035	4.17519175616998e-05	0.001597	0	0	0.005978	0.030248	0.004732	0.031692	6.35959852635787e-05	0.000292
Optum	late	25%	5	-0.00217	0.004555	-0.00077	0.002005	0	0	0.011953	0.02876	0.009462	0.031445	0.000132	0.000266
PharMetrics	early	10%	6	0.00023	0.002133	0.000291	0.001233	0	0	-0.00939	0.032803	-0.00739	0.017227	-9.40E-05	0.00033
PharMetrics	early	15%	6	-0.00136	0.003188	-0.0007	0.00149	0	0.09375	-0.01611	0.026509	-0.0158	0.020985	-0.00016	0.000297
PharMetrics	early	20%	6	-0.00167	0.002233	-0.00088	0.0015	0	0.075	-0.01506	0.035419	-0.00885	0.029417	-0.00014	0.000379
PharMetrics	early	25%	6	-0.0018	0.004187	-0.00094	0.005127	0	0	-0.01907	0.047115	-0.01135	0.061777	-0.00017	0.000483
PharMetrics	late	10%	5	0.000	0.003	0.000215	0.001	0	0	0.00531	0.018484	0.00307	0.018638	5.5433829727	0.00182

				446	959		954							683e-06	
PharMetrics	late	15%	5	0.000391	0.006117	0.000188	0.003048	0	0	0.003259	0.036581	0.001882	0.038906	3.54144859999878e-05	0.00357
PharMetrics	late	20%	5	-0.00056	0.007398	-0.00027	0.003493	0	0	0.003912	0.045113	0.002259	0.052629	4.33078547155408e-05	0.00438
PharMetrics	late	25%	5	-0.00267	0.008321	-0.00129	0.00383	0	0	0.009622	0.056341	0.005557	0.073252	0.000108	0.00539

IQR: Interquartile range.
