# Supplementary material for: Estimating cumulative incidence from partially missing time series of respiratory viral infections
Source: PLoS One. 2026 Sep 15;21(9):e0353681. doi: 10.1371/journal.pone.0353681 (PMC13577586; doi:10.1371/journal.pone.0353681)
Supplement: Supplementary file 7 [file pone.0353681.s007.pdf]

S5 Table. Season-by-season stability (P = 52.18)

**Data source**	**Season**	**Applicable scenarios**, n	**Surveillance coefficient (standardized)**, median	**Surveillance coefficient (standardized)**, IQR	**Model R^2**, median	**Model R^2**, IQR	**Residual SD**, median	**Residual SD**, IQR	**Pearson correlation (study vs surveillance, training overlap)**, median	**Pearson correlation (study vs surveillance, training overlap)**, IQR	**Spearman correlation (study vs surveillance, training overlap)**, median	**Spearman correlation (study vs surveillance, training overlap)**, IQR
KPNW	2021-22	7	2.377802	0.152691	0.856785	0.00902	1.295508	0.02949	0.903146	0.00687	0.696026	0.256629
KPNW	2022-23	5	7.542948	0.113183	0.810796	0.004423	4.515234	0.212596	0.891673	0.005287	0.920813	0.02324
KPNW	2023-24	7	5.302974	0.170166	0.889113	0.006123	1.947965	0.073728	0.941113	0.005854	0.952102	0.053359
Optum	2018-19	8	1.915953	0.087772	0.915852	0.02341	0.53548	0.017136	0.953496	0.016492	0.939016	0.008835
Optum	2019-20	8	2.630645	0.13975	0.941867	0.00544	0.543788	0.025351	0.961792	0.004066	0.956076	0.007353
Optum	2020-21	6	0.625208	0.126227	0.902847	0.003063	0.250516	0.021299	0.943438	0.003218	0.867977	0.064598
Optum	2021-22	8	0.981666	0.058815	0.786968	0.021811	0.644341	0.022017	0.878441	0.007954	0.714822	0.131872
Optum	2022-23	8	3.512067	0.145481	0.866441	0.010076	1.436227	0.104163	0.927329	0.004137	0.96007	0.037995
Optum	2023-24	8	7.224342	0.229909	0.893627	0.012263	2.378057	0.101263	0.943046	0.008052	0.973565	0.031555
PharMetrics	2018-19	8	1.441507	0.068497	0.868631	0.061117	0.507188	0.059859	0.923292	0.026569	0.934009	0.024944

PharMetrics	2019-20	8	1.908028	0.145712	0.881283	0.012187	0.6249	0.028391	0.936259	0.008229	0.936724	0.004727
PharMetrics	2020-21	4	0.582229	0.030044	0.882554	0.00223	0.229288	0.004545	0.929492	0.001753	0.84994	0.028393
PharMetrics	2021-22	8	0.577212	0.085876	0.817395	0.04804	0.479675	0.047039	0.864138	0.008783	0.775991	0.111451
PharMetrics	2022-23	8	2.857223	0.18294	0.931314	0.009743	0.863251	0.055378	0.961889	0.007655	0.940819	0.052437
PharMetrics	2023-24	8	7.799174	0.755702	0.906939	0.012665	2.165798	0.090466	0.941915	0.004347	0.974693	0.01801

IQR: Interquartile range; SD: Standard deviation.
