# Supplementary material for: Estimating cumulative incidence from partially missing time series of respiratory viral infections
Source: PLoS One. 2026 Sep 15;21(9):e0353681. doi: 10.1371/journal.pone.0353681 (PMC13577586; doi:10.1371/journal.pone.0353681)
Supplement: Supplementary file 8 [file pone.0353681.s008.pdf]

S6 Table. Correlation diagnostics vs error

**Data source**	**Missingness location** (early/late)	**Missingness level** (% of weeks omitted)	**Overlap correlation bin** (training overlap)	**Scenarios evaluated**, n	**Applicable scenarios**, n	**Applicability rate Cumulative percent error (%)**, median	**Cumulative percent error (%)**, median	**Cumulative percent error (%)**, IQR	**Cumulative signed difference (per 100,000)**, median	**Cumulative signed difference (per 100,000)**, IQR
KPNW	early	10%	≥0.7	3	3	1	-0.07046	0.938383	-0.27652	1.522422
KPNW	early	15%	≥0.7	2	2	1	0.169927	1.2963	-0.21462	2.425799
KPNW	early	15%	NA	1	0	0	NA	NA	NA	NA
KPNW	early	20%	≥0.7	2	2	1	-7.51799	0.302417	-14.607	3.725389
KPNW	early	20%	NA	1	0	0	NA	NA	NA	NA
KPNW	early	25%	NA	3	0	0	NA	NA	NA	NA
KPNW	late	10%	≥0.7	3	3	1	2.965299	0.681859	6.240425	5.664181
KPNW	late	15%	≥0.7	3	3	1	6.751651	1.303872	10.51596	8.132631
KPNW	late	20%	≥0.7	3	3	1	8.715466	1.579925	13.75345	11.14235
KPNW	late	25%	≥0.7	3	3	1	12.18178	2.983122	21.89339	13.88236
Optum	early	10%	≥0.7	6	6	1	1.357499	2.212179	1.278172	1.902455
Optum	early	15%	≥0.7	6	6	1	3.259308	3.591126	2.749913	2.582106
Optum	early	20%	≥0.7	6	6	1	5.126199	6.914404	5.959472	4.64208
Optum	early	25%	≥0.7	6	6	1	13.01987	5.833635	11.64414	22.8186
Optum	late	10%	≥0.7	6	6	1	0.438113	2.837298	0.328137	2.884932
Optum	late	15%	≥0.7	6	6	1	-0.26997	2.485892	-0.52185	3.72422

Optum	late	20%	≥0.7	5	5	1	-1.10332	1.297338	-2.05615	4.063306
Optum	late	20%	NA	1	0	0	NA	NA	NA	NA
Optum	late	25%	≥0.7	5	5	1	-2.36981	6.860793	-5.90469	2.901727
Optum	late	25%	NA	1	0	0	NA	NA	NA	NA
PharMetrics	early	10%	≥0.7	6	6	1	-0.82273	4.496946	-0.89022	3.769105
PharMetrics	early	15%	≥0.7	6	6	1	0.242534	9.338632	-1.24272	9.74202
PharMetrics	early	20%	≥0.7	6	6	1	3.88712	9.896303	2.621059	10.34213
PharMetrics	early	25%	≥0.7	6	6	1	10.75768	9.462599	5.270388	3.966019
PharMetrics	late	10%	≥0.7	5	5	1	1.894728	2.73603	2.748614	4.621842
PharMetrics	late	10%	NA	1	0	0	NA	NA	NA	NA
PharMetrics	late	15%	≥0.7	5	5	1	2.905988	6.155026	4.215612	8.441745
PharMetrics	late	15%	NA	1	0	0	NA	NA	NA	NA
PharMetrics	late	20%	≥0.7	5	5	1	2.938383	14.04832	4.262607	13.08378
PharMetrics	late	20%	NA	1	0	0	NA	NA	NA	NA
PharMetrics	late	25%	≥0.7	5	5	1	3.490397	22.6522	5.063393	17.2337
PharMetrics	late	25%	NA	1	0	0	NA	NA	NA	NA

IQR: Interquartile range; NA: Not applicable.
