# Supplementary material for: Estimating cumulative incidence from partially missing time series of respiratory viral infections
Source: PLoS One. 2026 Sep 15;21(9):e0353681. doi: 10.1371/journal.pone.0353681 (PMC13577586; doi:10.1371/journal.pone.0353681)
Supplement: Supplementary file 9 [file pone.0353681.s009.pdf]

S7 Table. Scenario-level results for all sources, seasons, and missingness scenarios (CSV). This machine-readable table contains one row per scenario (data source × season × missingness location × missingness level), including model coefficients, overlap diagnostics, prediction-interval summaries, and seasonal reconstruction error metrics. Scenarios classified as not applicable due to peak overlap are retained and flagged. The full table is provided as **S7 Table.csv**; Column definitions are listed below:

Column name	Definition
source	Data source
season	Season
gap	Missingness location (early/late)
miss_level	Missingness level (% of weeks omitted)
P	Harmonic period (P)
T_season	Season length (weeks)
n_missing	Weeks omitted, n
n_train	Training weeks, n
n_holdout	Holdout weeks, n
applicable	Applicable scenario
not_applicable_reason	Not applicable reason
peak_overlap_1	Peak overlap (max week)
peak_overlap_top3	Peak overlap (top 3 weeks)
prop_cases_missing	Proportion of seasonal cases missing (%)
corr_yx_train	Overlap correlation (Pearson; training)
rho_yx_train	Overlap correlation (Spearman; training)
beta0	Intercept (β0)
beta_xstd	Surveillance coefficient (standardized; β1)
beta_sine	Seasonal sine coefficient (β2)
beta_cosine	Seasonal cosine coefficient (β3)
r2	Model R^2
sigma_hat	Residual SD (σ)
rmse_holdout	Holdout RMSE
nrmse_holdout	Holdout nRMSE
neg_pred_count	Negative predictions, n
neg_pred_prop	Negative predictions, proportion
ljung_box_p	Ljung–Box p-value
ljung_box_lag	Ljung–Box lag
pi_coverage95	Empirical 95% PI coverage
pi_mean_width	Mean 95% PI width
cum_observed	Observed seasonal cumulative incidence
cum_incomplete	Incomplete seasonal cumulative incidence
cum_reconstructed	Reconstructed seasonal cumulative incidence
cum_percent_error	Cumulative percent error (%)
cum_signed_diff	Cumulative signed difference (per 100,000)
cum_log_ratio_error	Cumulative log-ratio error
