# Supplementary material for: Estimating cumulative incidence from partially missing time series of respiratory viral infections
Source: PLoS One. 2026 Sep 15;21(9):e0353681. doi: 10.1371/journal.pone.0353681 (PMC13577586; doi:10.1371/journal.pone.0353681)
Supplement: Supplementary file 10 [file pone.0353681.s010.pdf]

S8 Table. Weekly reconstruction performance and applicability for RSV clinical incidence (P = 52.18), sensitivity analysis excluding the 2023-24 season

**Data source**	**Missingness location** (Early-season / Late-season)	**Missingness level** (10%, 15%, 20%, 25% of weeks)	**Seasons evaluated** (n)	**Applicable scenarios** (n)	**Applicability rate**	**Weekly RMSE**, median (IQR)	**Weekly nRMSE**, median (IQR)	**Empirical 95% PI coverage**, median (IQR)
KPNW	early	10%	4	4	1	2.299 (2.092)	0.438 (0.011)	1.000 (0.000)
KPNW	early	15%	4	2	0.5	1.297 (0.001)	0.447 (0.000)	0.875 (0.000)
KPNW	early	20%	4	2	0.5	2.243 (0.002)	0.773 (0.001)	0.650 (0.050)
KPNW	early	25%	4	0	0	NA	NA	NA
KPNW	late	10%	4	4	1	2.139 (2.099)	0.399 (0.047)	1.000 (0.000)
KPNW	late	15%	4	4	1	2.421 (1.975)	0.472 (0.040)	0.938 (0.156)
KPNW	late	20%	4	4	1	2.562 (2.210)	0.493 (0.013)	0.950 (0.100)
KPNW	late	25%	4	4	1	2.853 (2.058)	0.571 (0.112)	0.923 (0.154)
Optum	early	10%	10	10	1	0.435 (0.290)	0.171 (0.069)	1.000 (0.000)
Optum	early	15%	10	10	1	0.609 (0.463)	0.265 (0.121)	0.838 (0.238)
Optum	early	20%	10	10	1	0.587 (0.413)	0.382 (0.345)	0.833 (0.545)
Optum	early	25%	10	10	1	1.119 (0.701)	0.739 (0.315)	0.481 (0.356)
Optum	late	10%	10	10	1	0.450 (0.166)	0.269 (0.177)	1.000 (0.200)
Optum	late	15%	10	10	1	0.413 (0.174)	0.244 (0.195)	0.900 (0.238)
Optum	late	20%	10	8	0.8	0.428 (0.277)	0.186 (0.163)	1.000 (0.117)
Optum	late	25%	10	8	0.8	0.829 (0.671)	0.349 (0.222)	0.712 (0.562)
PharMetrics	early	10%	10	10	1	0.592 (0.089)	0.271 (0.056)	1.000 (0.200)
PharMetrics	early	15%	10	10	1	0.852 (0.264)	0.425 (0.180)	0.812 (0.394)
PharMetrics	early	20%	10	10	1	0.904 (0.210)	0.500 (0.156)	0.667 (0.033)
PharMetrics	early	25%	10	10	1	0.968 (0.183)	0.637 (0.235)	0.500 (0.240)
PharMetrics	late	10%	10	8	0.8	0.712 (0.404)	0.347 (0.345)	0.800 (0.467)
PharMetrics	late	15%	10	8	0.8	0.726 (0.460)	0.413 (0.438)	0.750 (0.719)
PharMetrics	late	20%	10	8	0.8	0.956 (0.237)	0.563 (0.271)	0.667 (0.533)
PharMetrics	late	25%	10	8	0.8	1.086 (0.541)	0.660 (0.173)	0.534 (0.495)

95% PI: 95% Prediction intervals; IQR: Interquartile range; nRMSE: Normalized root mean squared error; RMSE: Root mean squared error; RSV: Respiratory syncytial virus.
