# Supplementary material for: Estimating cumulative incidence from partially missing time series of respiratory viral infections
Source: PLoS One. 2026 Sep 15;21(9):e0353681. doi: 10.1371/journal.pone.0353681 (PMC13577586; doi:10.1371/journal.pone.0353681)
Supplement: Supplementary file 11 [file pone.0353681.s011.pdf]

S9 Table. Seasonal cumulative reconstruction accuracy for RSV clinical incidence under early-season missingness (P = 52.18), sensitivity analysis excluding the 2023–24 season

Data Source	Missingness level (10%, 15%, 20%, 25% of weeks)	Seasons evaluated (n)	Applicable scenarios (n)	Applicability rate	Cumulative percent error (%), median (IQR)	Cumulative signed difference (per 100,000), median (IQR)	Cumulative log-ratio error, median (IQR)
KPNW	10%	4	4	1	-0.87 (1.52)	-1.46 (2.03)	-0.009 (0.015)
KPNW	15%	4	2	0.5	1.43 (0.04)	2.15 (0.06)	0.014 (0.000)
KPNW	20%	4	2	0.5	-7.24 (0.03)	-10.92 (0.04)	-0.075 (0.000)
KPNW	25%	4	0	0	NA	NA	NA
Optum	10%	10	10	1	1.64 (1.10)	1.30 (0.64)	0.016 (0.011)
Optum	15%	10	10	1	3.45 (1.90)	2.83 (1.53)	0.034 (0.018)
Optum	20%	10	10	1	7.09 (7.92)	5.61 (3.58)	0.068 (0.074)
Optum	25%	10	10	1	12.33 (7.35)	10.18 (4.82)	0.116 (0.064)
PharMetrics	10%	10	10	1	-0.54 (3.74)	-0.16 (3.15)	-0.005 (0.037)
PharMetrics	15%	10	10	1	2.70 (8.94)	0.79 (7.50)	0.027 (0.088)
PharMetrics	20%	10	10	1	4.40 (3.35)	4.26 (4.96)	0.043 (0.032)
PharMetrics	25%	10	10	1	11.83 (5.13)	6.22 (2.56)	0.112 (0.046)

IQR: Interquartile range; NA: Not applicable; RSV: Respiratory syncytial virus.
