# Supplementary material for: Estimating cumulative incidence from partially missing time series of respiratory viral infections
Source: PLoS One. 2026 Sep 15;21(9):e0353681. doi: 10.1371/journal.pone.0353681 (PMC13577586; doi:10.1371/journal.pone.0353681)
Supplement: Supplementary file 12 [file pone.0353681.s012.pdf]

S10 Table. Seasonal cumulative reconstruction accuracy for RSV clinical incidence under late-season missingness (P = 52.18), sensitivity analysis excluding the 2023-24 season

Data Source	Missingness level (10%, 15%, 20%, 25% of weeks)	Seasons evaluated (n)	Applicable scenarios (n)	Applicability rate	Cumulative percent error (%), median (IQR)	Cumulative signed difference (per 100,000), median (IQR)	Cumulative log-ratio error, median (IQR)
KPNW	10%	4	4	1	3.51 (1.04)	10.16 (11.30)	0.035 (0.010)
KPNW	15%	4	4	1	6.88 (0.22)	18.56 (15.93)	0.067 (0.002)
KPNW	20%	4	4	1	8.92 (0.25)	24.36 (22.22)	0.085 (0.002)
KPNW	25%	4	4	1	13.38 (2.31)	34.92 (25.87)	0.126 (0.020)
Optum	10%	10	10	1	0.99 (2.13)	0.86 (1.43)	0.010 (0.021)
Optum	15%	10	10	1	0.04 (1.84)	0.02 (2.16)	0.000 (0.018)
Optum	20%	10	8	0.8	-0.93 (2.69)	-1.34 (3.22)	-0.009 (0.027)
Optum	25%	10	8	0.8	-5.69 (8.67)	-5.86 (5.64)	-0.059 (0.091)
PharMetrics	10%	10	8	0.8	0.60 (4.41)	1.15 (4.06)	0.006 (0.044)
PharMetrics	15%	10	8	0.8	0.19 (8.40)	1.30 (7.27)	0.002 (0.084)
PharMetrics	20%	10	8	0.8	-1.75 (12.51)	0.06 (9.99)	-0.019 (0.126)
PharMetrics	25%	10	8	0.8	-3.37 (16.74)	-0.53 (12.88)	-0.037 (0.170)

IQR: Interquartile range; RSV: Respiratory syncytial virus.
