# Supplementary material for: Estimating cumulative incidence from partially missing time series of respiratory viral infections
Source: PLoS One. 2026 Sep 15;21(9):e0353681. doi: 10.1371/journal.pone.0353681 (PMC13577586; doi:10.1371/journal.pone.0353681)
Supplement: Supplementary file 13 [file pone.0353681.s013.pdf]

**S11 Table. Sensitivity analysis using non-negative link (quasi-Poisson/gamma-log) for early ≈20% gaps: Comparison of Ordinary Least Squares and Gamma Generalized Linear Model reconstructed cumulative RSV incidence, percent errors, and deltas by source and season**

| **Source** | **Season** | **Number of weeks missing** (≈20% of the season) | **Observed full-season cumulative incidence** (per 100,000) | **Cumulative incidence of the observed** (non-omitted) weeks (per 100,000) | **Reconstructed cumulative incidence using OLS** (per 100,000) | **Reconstructed cumulative incidence using non-negative GLM** (per 100,000) | **Percent error of OLS reconstruction vs total true** | **Percent error of GLM reconstruction vs total true** | **GLM − OLS difference in reconstructed total** (per 100,000) | **GLM − OLS difference in percent error** (percentage points) |
|---|---|---|---|---|---|---|---|---|---|---|
| KPNW | 2122 | 10 | 150.81 | 88.53 | 139.85 | 124.09 | -7.27 | -17.72 | -15.76 | -10.45 |
| KPNW | 2122 | 10 | 150.81 | 88.53 | 139.93 | 124.09 | -7.22 | -17.72 | -15.84 | -10.50 |
| KPNW | 2223 | 10 | 392.47 | 183.22 | NA | 349.02 | NA | -11.07 | NA | NA |
| KPNW | 2324 | 10 | 234.42 | 157.14 | 216.12 | 185.40 | -7.81 | -20.91 | -30.71 | -13.10 |
| KPNW | 2324 | 10 | 234.42 | 157.14 | 216.09 | 185.40 | -7.82 | -20.91 | -30.68 | -13.09 |
| Optum | 1819 | 6 | 79.16 | 75.05 | 84.76 | 80.81 | 7.08 | 2.09 | -3.95 | -4.99 |
| Optum | 1819 | 6 | 79.16 | 75.05 | 84.77 | 80.81 | 7.10 | 2.09 | -3.96 | -5.01 |
| Optum | 1920 | 6 | 87.31 | 81.11 | 90.03 | 85.71 | 3.12 | -1.83 | -4.33 | -4.96 |
| Optum | 1920 | 6 | 87.31 | 81.11 | 90.04 | 85.71 | 3.12 | -1.83 | -4.33 | -4.96 |
| Optum | 2021 | 11 | 57.13 | 53.17 | 63.44 | 62.36 | 11.04 | 9.15 | -1.08 | -1.89 |
| Optum | 2021 | 11 | 57.13 | 53.17 | 63.43 | 62.36 | 11.03 | 9.15 | -1.07 | -1.88 |
| Optum | 2122 | 10 | 106.35 | 72.11 | 105.01 | 105.78 | -1.26 | -0.54 | 0.77 | 0.72 |
| Optum | 2122 | 10 | 106.35 | 72.11 | 105.02 | 105.78 | -1.25 | -0.54 | 0.75 | 0.71 |
| Optum | 2223 | 10 | 186.36 | 115.14 | 210.16 | 216.07 | 12.77 | 15.94 | 5.91 | 3.17 |
| Optum | 2223 | 10 | 186.36 | 115.14 | 210.11 | 216.07 | 12.74 | 15.94 | 5.96 | 3.20 |
| Optum | 2324 | 10 | 275.36 | 210.47 | 284.19 | 256.04 | 3.21 | -7.01 | -28.15 | -10.22 |
| Optum | 2324 | 10 | 275.36 | 210.47 | 284.05 | 256.04 | 3.16 | -7.01 | -28.00 | -10.17 |
| PharMetrics | 1819 | 6 | 57.75 | 54.99 | 63.65 | 60.31 | 10.21 | 4.42 | -3.34 | -5.79 |
| PharMetrics | 1819 | 6 | 57.75 | 54.99 | 63.66 | 60.31 | 10.23 | 4.42 | -3.36 | -5.81 |
| PharMetrics | 1920 | 6 | 64.31 | 61.69 | 68.56 | 64.94 | 6.62 | 0.98 | -3.62 | -5.63 |
| PharMetrics | 1920 | 6 | 64.31 | 61.69 | 68.56 | 64.94 | 6.62 | 0.98 | -3.62 | -5.64 |
| PharMetrics | 2021 | 11 | 29.10 | 26.26 | 30.03 | 29.61 | 3.22 | 1.76 | -0.43 | -1.46 |
| PharMetrics | 2021 | 11 | 29.10 | 26.26 | 30.08 | 29.61 | 3.39 | 1.76 | -0.47 | -1.62 |

| | | | | | | | | | | |
|---|---|---|---|---|---|---|---|---|---|---|
| PharMetrics | 2122 | 10 | 73.87 | 44.60 | 67.05 | 67.89 | -9.23 | -8.09 | 0.84 | 1.14 |
| PharMetrics | 2122 | 10 | 73.87 | 44.60 | 67.08 | 67.89 | -9.19 | -8.09 | 0.81 | 1.10 |
| PharMetrics | 2223 | 10 | 145.07 | 80.52 | 151.46 | 146.99 | 4.41 | 1.33 | -4.47 | -3.08 |
| PharMetrics | 2223 | 10 | 145.07 | 80.52 | 151.43 | 146.99 | 4.39 | 1.33 | -4.44 | -3.06 |
| PharMetrics | 2324 | 7 | 233.02 | 211.71 | 218.48 | 236.96 | -6.24 | 1.69 | 18.47 | 7.93 |
| PharMetrics | 2324 | 7 | 233.02 | 211.71 | 218.48 | 236.96 | -6.24 | 1.69 | 18.48 | 7.93 |

RSV: Respiratory syncytial virus.
