# Supplementary material for: Estimating cumulative incidence from partially missing time series of respiratory viral infections
Source: PLoS One. 2026 Sep 15;21(9):e0353681. doi: 10.1371/journal.pone.0353681 (PMC13577586; doi:10.1371/journal.pone.0353681)
Supplement: Supplementary file 14 [file pone.0353681.s014.pdf]

S12 Table. Incomplete vs reconstructed vs observed cumulative RSV incidence by scenario (source × season × side × missingness)

Data source	RSV season	Gap location (early or late)	Proportion of season removed	Number of weeks removed in the gap	Incomplete cumulative incidence (no imputation; per 100,000)	Reconstructed cumulative incidence (model; per 100,000)	Observed cumulative incidence (full season; per 100,000)
KPNW	2122	early	10%	5	125.30	148.30	150.81
KPNW	2122	early	10%	5	125.30	148.36	150.81
KPNW	2122	early	15%	8	106.84	152.90	150.81
KPNW	2122	early	15%	8	106.84	153.02	150.81
KPNW	2122	early	20%	10	88.53	139.85	150.81
KPNW	2122	early	20%	10	88.53	139.93	150.81
KPNW	2122	early	25%	13	54.57	NA	150.81
KPNW	2122	early	25%	13	54.57	NA	150.81
KPNW	2122	late	10%	5	144.26	155.33	150.81
KPNW	2122	late	10%	5	144.26	155.28	150.81
KPNW	2122	late	15%	8	142.08	161.43	150.81
KPNW	2122	late	15%	8	142.08	161.32	150.81
KPNW	2122	late	20%	10	140.67	164.09	150.81
KPNW	2122	late	20%	10	140.67	163.95	150.81
KPNW	2122	late	25%	13	138.02	172.84	150.81
KPNW	2122	late	25%	13	138.02	172.70	150.81
KPNW	2223	early	10%	5	342.27	392.00	392.47
KPNW	2223	early	10%	5	342.27	392.20	392.47
KPNW	2223	early	15%	8	237.17	NA	392.47
KPNW	2223	early	15%	8	237.17	NA	392.47
KPNW	2223	early	20%	10	183.22	NA	392.47
KPNW	2223	early	20%	10	183.22	NA	392.47
KPNW	2223	early	25%	13	131.45	NA	392.47
KPNW	2223	early	25%	13	131.45	NA	392.47
KPNW	2223	late	10%	5	383.89	408.31	392.47
KPNW	2223	late	10%	5	383.89	408.27	392.47
KPNW	2223	late	15%	8	377.95	419.09	392.47
KPNW	2223	late	15%	8	377.95	418.97	392.47
KPNW	2223	late	20%	10	374.83	428.07	392.47
KPNW	2223	late	20%	10	374.83	427.90	392.47
KPNW	2223	late	25%	13	369.68	440.52	392.47
KPNW	2223	late	25%	13	369.68	440.28	392.47
KPNW	2324	early	10%	5	216.46	234.96	234.42
KPNW	2324	early	10%	5	216.46	235.02	234.42
KPNW	2324	early	15%	8	186.96	231.71	234.42
KPNW	2324	early	15%	8	186.96	231.78	234.42
KPNW	2324	early	20%	10	157.14	216.12	234.42
KPNW	2324	early	20%	10	157.14	216.09	234.42
KPNW	2324	early	25%	13	101.56	NA	234.42
KPNW	2324	early	25%	13	101.56	NA	234.42
KPNW	2324	late	10%	5	232.00	240.65	234.42

KPNW	2324	late	10%	5	232.00	240.66	234.42
KPNW	2324	late	15%	8	230.06	244.66	234.42
KPNW	2324	late	15%	8	230.06	244.65	234.42
KPNW	2324	late	20%	10	228.94	248.17	234.42
KPNW	2324	late	20%	10	228.94	248.17	234.42
KPNW	2324	late	25%	13	227.00	254.44	234.42
KPNW	2324	late	25%	13	227.00	254.46	234.42
Optum	1819	early	10%	3	77.34	80.45	79.16
Optum	1819	early	10%	3	77.34	80.46	79.16
Optum	1819	early	15%	5	75.93	83.35	79.16
Optum	1819	early	15%	5	75.93	83.35	79.16
Optum	1819	early	20%	6	75.05	84.76	79.16
Optum	1819	early	20%	6	75.05	84.77	79.16
Optum	1819	early	25%	8	73.14	87.44	79.16
Optum	1819	early	25%	8	73.14	87.46	79.16
Optum	1819	late	10%	3	75.24	76.89	79.16
Optum	1819	late	10%	3	75.24	76.89	79.16
Optum	1819	late	15%	5	71.92	75.67	79.16
Optum	1819	late	15%	5	71.92	75.67	79.16
Optum	1819	late	20%	6	69.49	74.40	79.16
Optum	1819	late	20%	6	69.49	74.40	79.16
Optum	1819	late	25%	8	63.92	71.85	79.16
Optum	1819	late	25%	8	63.92	71.84	79.16
Optum	1920	early	10%	3	84.36	89.20	87.31
Optum	1920	early	10%	3	84.36	89.20	87.31
Optum	1920	early	15%	5	82.16	89.98	87.31
Optum	1920	early	15%	5	82.16	89.98	87.31
Optum	1920	early	20%	6	81.11	90.03	87.31
Optum	1920	early	20%	6	81.11	90.04	87.31
Optum	1920	early	25%	8	77.98	88.43	87.31
Optum	1920	early	25%	8	77.98	88.43	87.31
Optum	1920	late	10%	3	85.42	88.17	87.31
Optum	1920	late	10%	3	85.42	88.17	87.31
Optum	1920	late	15%	5	83.80	88.43	87.31
Optum	1920	late	15%	5	83.80	88.43	87.31
Optum	1920	late	20%	6	81.93	86.61	87.31
Optum	1920	late	20%	6	81.93	86.62	87.31
Optum	1920	late	25%	8	74.91	79.45	87.31
Optum	1920	late	25%	8	74.91	79.45	87.31
Optum	2021	early	10%	5	55.45	58.39	57.13
Optum	2021	early	10%	5	55.45	58.39	57.13
Optum	2021	early	15%	8	54.31	59.96	57.13
Optum	2021	early	15%	8	54.31	59.96	57.13
Optum	2021	early	20%	11	53.17	63.44	57.13
Optum	2021	early	20%	11	53.17	63.43	57.13
Optum	2021	early	25%	13	52.33	67.32	57.13
Optum	2021	early	25%	13	52.33	67.31	57.13
Optum	2021	late	10%	5	45.41	58.37	57.13
Optum	2021	late	10%	5	45.41	58.36	57.13
Optum	2021	late	15%	8	37.85	57.16	57.13

Optum	2021	late	15%	8	37.85	57.15	57.13
Optum	2021	late	20%	11	30.19	NA	57.13
Optum	2021	late	20%	11	30.19	NA	57.13
Optum	2021	late	25%	13	26.29	NA	57.13
Optum	2021	late	25%	13	26.29	NA	57.13
Optum	2122	early	10%	5	91.98	105.71	106.35
Optum	2122	early	10%	5	91.98	105.72	106.35
Optum	2122	early	15%	8	81.62	104.57	106.35
Optum	2122	early	15%	8	81.62	104.58	106.35
Optum	2122	early	20%	10	72.11	105.01	106.35
Optum	2122	early	20%	10	72.11	105.02	106.35
Optum	2122	early	25%	13	61.30	119.46	106.35
Optum	2122	early	25%	13	61.30	119.46	106.35
Optum	2122	late	10%	5	97.55	108.50	106.35
Optum	2122	late	10%	5	97.55	108.49	106.35
Optum	2122	late	15%	8	93.14	109.39	106.35
Optum	2122	late	15%	8	93.14	109.36	106.35
Optum	2122	late	20%	10	90.60	110.44	106.35
Optum	2122	late	20%	10	90.60	110.41	106.35
Optum	2122	late	25%	13	86.66	112.13	106.35
Optum	2122	late	25%	13	86.66	112.10	106.35
Optum	2223	early	10%	5	164.57	188.34	186.36
Optum	2223	early	10%	5	164.57	188.36	186.36
Optum	2223	early	15%	8	138.88	192.77	186.36
Optum	2223	early	15%	8	138.88	192.81	186.36
Optum	2223	early	20%	10	115.14	210.16	186.36
Optum	2223	early	20%	10	115.14	210.11	186.36
Optum	2223	early	25%	13	87.62	257.75	186.36
Optum	2223	early	25%	13	87.62	257.57	186.36
Optum	2223	late	10%	5	178.95	186.20	186.36
Optum	2223	late	10%	5	178.95	186.16	186.36
Optum	2223	late	15%	8	175.97	185.36	186.36
Optum	2223	late	15%	8	175.97	185.30	186.36
Optum	2223	late	20%	10	174.35	184.38	186.36
Optum	2223	late	20%	10	174.35	184.30	186.36
Optum	2223	late	25%	13	171.74	182.03	186.36
Optum	2223	late	25%	13	171.74	181.94	186.36
Optum	2324	early	10%	5	258.47	269.72	275.36
Optum	2324	early	10%	5	258.47	269.59	275.36
Optum	2324	early	15%	8	237.69	276.32	275.36
Optum	2324	early	15%	8	237.69	276.17	275.36
Optum	2324	early	20%	10	210.47	284.19	275.36
Optum	2324	early	20%	10	210.47	284.05	275.36
Optum	2324	early	25%	13	159.15	313.42	275.36
Optum	2324	early	25%	13	159.15	313.11	275.36
Optum	2324	late	10%	5	271.48	271.48	275.36
Optum	2324	late	10%	5	271.48	271.49	275.36
Optum	2324	late	15%	8	269.45	270.29	275.36
Optum	2324	late	15%	8	269.45	270.31	275.36
Optum	2324	late	20%	10	268.21	269.56	275.36

Optum	2324	late	20%	10	268.21	269.60	275.36
Optum	2324	late	25%	13	266.02	269.39	275.36
Optum	2324	late	25%	13	266.02	269.45	275.36
PharMetrics	1819	early	10%	3	56.65	59.26	57.75
PharMetrics	1819	early	10%	3	56.65	59.26	57.75
PharMetrics	1819	early	15%	5	55.61	61.95	57.75
PharMetrics	1819	early	15%	5	55.61	61.96	57.75
PharMetrics	1819	early	20%	6	54.99	63.65	57.75
PharMetrics	1819	early	20%	6	54.99	63.66	57.75
PharMetrics	1819	early	25%	8	53.09	64.58	57.75
PharMetrics	1819	early	25%	8	53.09	64.59	57.75
PharMetrics	1819	late	10%	3	54.96	55.36	57.75
PharMetrics	1819	late	10%	3	54.96	55.36	57.75
PharMetrics	1819	late	15%	5	52.25	53.14	57.75
PharMetrics	1819	late	15%	5	52.25	53.14	57.75
PharMetrics	1819	late	20%	6	50.69	52.17	57.75
PharMetrics	1819	late	20%	6	50.69	52.16	57.75
PharMetrics	1819	late	25%	8	47.62	51.63	57.75
PharMetrics	1819	late	25%	8	47.62	51.63	57.75
PharMetrics	1920	early	10%	3	63.30	66.01	64.31
PharMetrics	1920	early	10%	3	63.30	66.01	64.31
PharMetrics	1920	early	15%	5	62.58	68.59	64.31
PharMetrics	1920	early	15%	5	62.58	68.59	64.31
PharMetrics	1920	early	20%	6	61.69	68.56	64.31
PharMetrics	1920	early	20%	6	61.69	68.56	64.31
PharMetrics	1920	early	25%	8	59.68	70.52	64.31
PharMetrics	1920	early	25%	8	59.68	70.53	64.31
PharMetrics	1920	late	10%	3	62.99	63.85	64.31
PharMetrics	1920	late	10%	3	62.99	63.86	64.31
PharMetrics	1920	late	15%	5	61.68	62.68	64.31
PharMetrics	1920	late	15%	5	61.68	62.68	64.31
PharMetrics	1920	late	20%	6	59.77	60.16	64.31
PharMetrics	1920	late	20%	6	59.77	60.16	64.31
PharMetrics	1920	late	25%	8	55.36	57.73	64.31
PharMetrics	1920	late	25%	8	55.36	57.73	64.31
PharMetrics	2021	early	10%	5	27.76	28.93	29.10
PharMetrics	2021	early	10%	5	27.76	28.94	29.10
PharMetrics	2021	early	15%	8	27.23	29.87	29.10
PharMetrics	2021	early	15%	8	27.23	29.90	29.10
PharMetrics	2021	early	20%	11	26.26	30.03	29.10
PharMetrics	2021	early	20%	11	26.26	30.08	29.10
PharMetrics	2021	early	25%	13	26.15	33.35	29.10
PharMetrics	2021	early	25%	13	26.15	33.42	29.10
PharMetrics	2021	late	10%	5	19.66	NA	29.10
PharMetrics	2021	late	10%	5	19.66	NA	29.10
PharMetrics	2021	late	15%	8	14.92	NA	29.10
PharMetrics	2021	late	15%	8	14.92	NA	29.10
PharMetrics	2021	late	20%	11	11.17	NA	29.10
PharMetrics	2021	late	20%	11	11.17	NA	29.10
PharMetrics	2021	late	25%	13	9.92	NA	29.10

PharMetrics	2021	late	25%	13	9.92	NA	29.10
PharMetrics	2122	early	10%	5	60.73	70.81	73.87
PharMetrics	2122	early	10%	5	60.73	70.84	73.87
PharMetrics	2122	early	15%	8	50.99	66.42	73.87
PharMetrics	2122	early	15%	8	50.99	66.45	73.87
PharMetrics	2122	early	20%	10	44.60	67.05	73.87
PharMetrics	2122	early	20%	10	44.60	67.08	73.87
PharMetrics	2122	early	25%	13	36.84	76.03	73.87
PharMetrics	2122	early	25%	13	36.84	76.05	73.87
PharMetrics	2122	late	10%	5	68.98	78.06	73.87
PharMetrics	2122	late	10%	5	68.98	78.04	73.87
PharMetrics	2122	late	15%	8	66.58	80.73	73.87
PharMetrics	2122	late	15%	8	66.58	80.69	73.87
PharMetrics	2122	late	20%	10	65.20	82.86	73.87
PharMetrics	2122	late	20%	10	65.20	82.81	73.87
PharMetrics	2122	late	25%	13	62.76	85.06	73.87
PharMetrics	2122	late	25%	13	62.76	84.98	73.87
PharMetrics	2223	early	10%	5	124.49	143.42	145.07
PharMetrics	2223	early	10%	5	124.49	143.44	145.07
PharMetrics	2223	early	15%	8	100.29	141.76	145.07
PharMetrics	2223	early	15%	8	100.29	141.78	145.07
PharMetrics	2223	early	20%	10	80.52	151.46	145.07
PharMetrics	2223	early	20%	10	80.52	151.43	145.07
PharMetrics	2223	early	25%	13	59.04	182.00	145.07
PharMetrics	2223	early	25%	13	59.04	181.89	145.07
PharMetrics	2223	late	10%	5	140.85	147.84	145.07
PharMetrics	2223	late	10%	5	140.85	147.82	145.07
PharMetrics	2223	late	15%	8	139.22	149.33	145.07
PharMetrics	2223	late	15%	8	139.22	149.28	145.07
PharMetrics	2223	late	20%	10	138.09	149.39	145.07
PharMetrics	2223	late	20%	10	138.09	149.33	145.07
PharMetrics	2223	late	25%	13	136.93	150.22	145.07
PharMetrics	2223	late	25%	13	136.93	150.13	145.07
PharMetrics	2324	early	10%	4	225.47	225.60	233.02
PharMetrics	2324	early	10%	4	225.47	225.60	233.02
PharMetrics	2324	early	15%	5	222.69	223.43	233.02
PharMetrics	2324	early	15%	5	222.69	223.43	233.02
PharMetrics	2324	early	20%	7	211.71	218.48	233.02
PharMetrics	2324	early	20%	7	211.71	218.48	233.02
PharMetrics	2324	early	25%	9	193.35	204.27	233.02
PharMetrics	2324	early	25%	9	193.35	204.21	233.02
PharMetrics	2324	late	10%	4	228.09	237.74	233.02
PharMetrics	2324	late	10%	4	228.09	237.77	233.02
PharMetrics	2324	late	15%	5	227.42	241.44	233.02
PharMetrics	2324	late	15%	5	227.42	241.48	233.02
PharMetrics	2324	late	20%	7	224.04	250.65	233.02
PharMetrics	2324	late	20%	7	224.04	250.74	233.02
PharMetrics	2324	late	25%	9	218.76	261.83	233.02
PharMetrics	2324	late	25%	9	218.76	261.97	233.02

NA: Not applicable; RSV: Respiratory syncytial virus.
